# Exclusive breastfeeding policy, practice and influences in South Africa, 1980 to 2018: A mixed-methods systematic review

**DOI:** 10.1371/journal.pone.0224029

**Published:** 2019-10-18

**Authors:** Sara Jewett Nieuwoudt, Christian B. Ngandu, Lenore Manderson, Shane A. Norris

**Affiliations:** 1 School of Public Health, Faculty of Health Sciences, University of the Witwatersrand, Braamfontein, Johannesburg, South Africa; 2 SAMRC Developmental Pathways Health and Research Unit, University of the Witwatersrand, Braamfontein, Johannesburg, South Africa; 3 Institute at Brown for Environment and Society, Brown University, Providence, RI, United States of America; 4 School of Social Sciences, Menzies Building, Clayton Campus, Monash University, Melbourne, Australia; 5 School of Human Development and Health, and NIHR Southampton Biomedical Research Centre, University of Southampton, Southampton, United Kingdom; Medical Research Council, SOUTH AFRICA

## Abstract

**Background:**

In 2011, South Africa committed to promoting exclusive breastfeeding (EBF) for six months for all mothers, regardless of HIV status, in line with World Health Organization recommendations. This was a marked shift from earlier policies, and with it, average EBF rates increased from less than 10% in 2011 to 32% by 2016.

**Objectives:**

The aim of this mixed-methods systematic review was to describe EBF practices in South Africa and their multi-level influences over four policy periods.

**Methods:**

We applied PRISMA guidelines according to a published protocol (Prospero: CRD42014010512). We searched seven databases [Africa-Wide, PubMed, Popline, PsychINFO, CINAHL, Global Health, and The Cochrane Library] and conducted hand searches for eligible articles (all study designs, conducted in South Africa and published between 1980–2018). The quality of articles was assessed using published tools, as appropriate. Separate policy analysis was conducted to delineate four distinct policy periods. We compared EBF rates by these periods. Then, applying a three-level ecological framework, we analysed EBF influences concurrently by method. Finally, the findings were synthesized to compare breastfeeding influences by policy period, maintaining an ecological framework.

**Results:**

From an initial sample of 20,226 articles, 72 unique articles were reviewed, three of which contributed to both quantitative and qualitative analysis. Despite the large sample, several provinces were poorly represented (if at all) and many studies were assessed as low to moderate quality. Despite these limitations, our historical lens enabled us to explore why South African progress on increasing EBF practices has been slow. The review reflects a context that increasingly supports EBF, but falls short in accounting for family, community, and workplace influences. The findings also highlight the unintended damage caused by rapidly adopting and introducing global guidelines to an unsupported health workforce.

**Conclusions:**

From a South African perspective, we identified geographic and methodological biases, as well as gaps in our understanding and potential explanations of inequities in EBF. Our recommendations relate to policy, programming, and research to inform changes that would be required to further improve EBF practice rates in South Africa. While our review is South Africa-specific, our findings have broader implications for investing in multi-level interventions and limiting how often infant feeding guidelines are changed.

## Introduction

Children depend on their families and communities to make dietary decisions on their behalf. The contexts of such decisions change over time, as do guidelines on what is the best for infants [1]. The current public health consensus is that exclusive breastfeeding (EBF) for the first six months is the best start for health and development [2]. The World Health Organization (WHO) defines EBF as infants consuming only breast milk, with the exception of oral rehydration solutions (ORS), drops or syrups [3]. EBF forms part of a broader definition of optimal infant and young child feeding (IYCF) practices, which also include initiation of breastfeeding within the first hour and continued breastfeeding for two years with the introduction of safe, adequate and appropriate complementary foods from six months [3]. A large volume of breastfeeding research has been conducted in South Africa, but this has not been systematically reviewed to identify factors that promote EBF. Identifying factors that promote (or inhibit) behavior is an essential step for the design of evidence-based policies and interventions that are sensitive to context [4].

Regrettably, scientific knowledge of the life-saving benefits of EBF has not translated into practice in South Africa. Globally, exclusively breastfed infant’s risk of death is 12% that of infants who are not breastfed [2]. The same authors estimate that universal EBF would avert an estimated 13.8% of deaths of children below age two. However, South Africa’s average EBF rate for infants below six months only recently rose to 32% [5] from rates closer to 7% in the 1998 national survey [6]. In fact, the national rates are even lower if one considers that only 23.7% of infants between four and five months were exclusively breastfed [5].

One explanation for South Africa’s low rates of EBF is the country’s high HIV prevalence, with 30.8% of mothers attending antenatal care testing HIV positive [7]. As epidemiologists tried to identify the risk of mother to child transmission early in the epidemic, government health facilities provided eligible HIV positive mothers with the option of receiving free commercial formula to support that choice [8, 9]. As antiretroviral therapy (ART) became more available and transmission risk dropped, South Africa’s Department of Health proclaimed the 2011 Tshwane Declaration for the promotion of breastfeeding and discontinued its free formula program [10].

**Fig 1:**
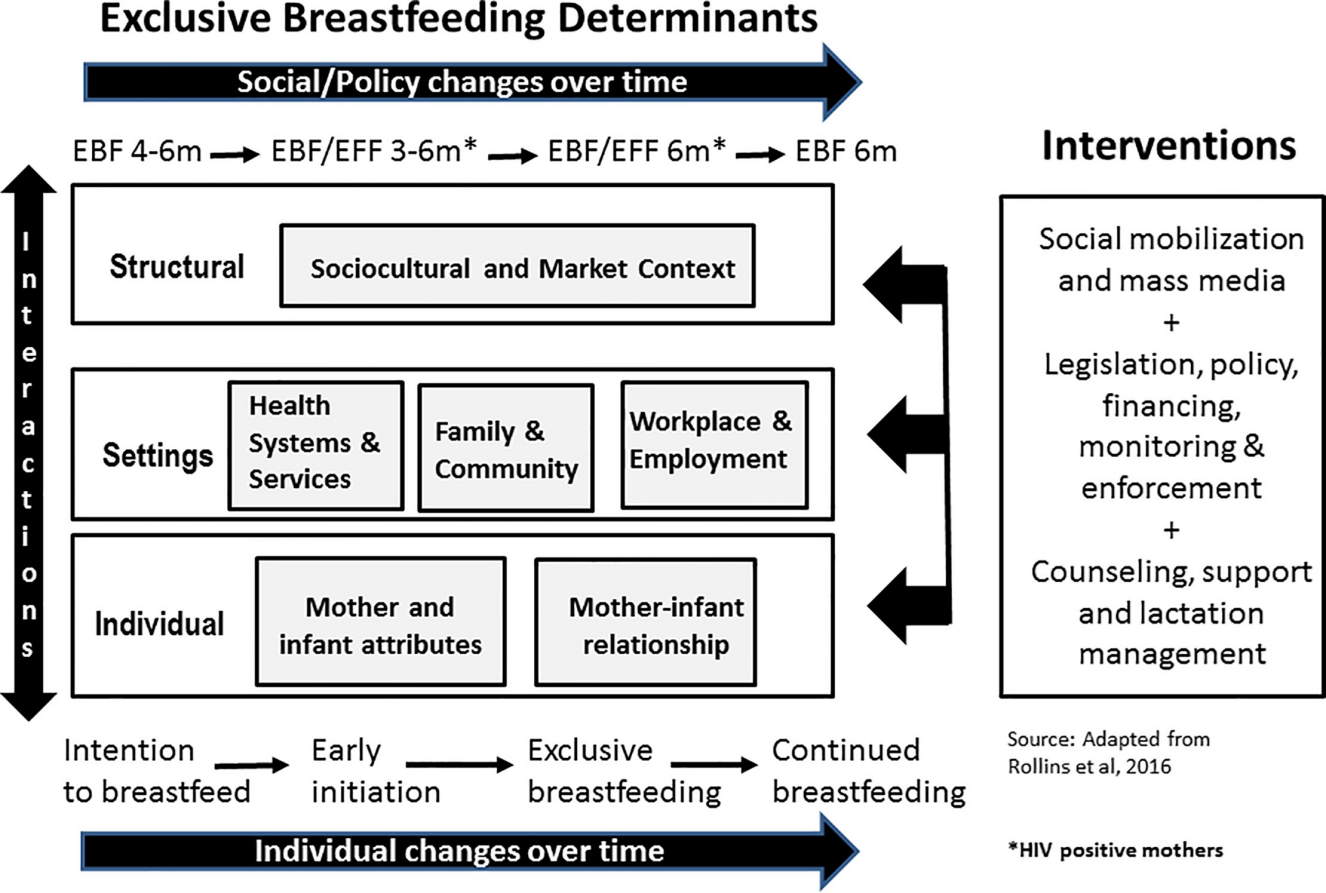
Adapted ecological model for breastfeeding determinants and interventions.

However, the HIV epidemic and free formula can be only part of the explanation for low EBF rates [11], with culturally established mixed feeding practices reported from well before the epidemic [12–14]. Consistent with an understanding that multiple factors shape infant feeding, scholars have argued for comprehensive interventions [4, 15]. Given the complex mix of factors that influence infant feeding, we adapted an ecological conceptual framework proposed by Rollins and colleagues [4] to synthesize and analyze factors that influence EBF. Our adaptation was to account for policy changes over time in addition to the original time element for the mother/infant dyad (see Fig 1).

As we were particularly interested in infant feeding guideline changes, this systematic review analyzed four critical policy periods, summarized in Table 1. These highlight policies before the pediatric HIV epidemic (Period 1), during the early years of the epidemic (Periods 2 and 3), and into the antiretroviral period (Period 4) when South Africa reverted to universal breastfeeding guidelines for all women. While the HIV epidemic became a lens for differentiating policy periods, national IYCF guidelines were also consulted as a way to reflect the broader policy environment.

**Table 1:** Infant feeding policy periods in South Africa.

Policy Period	Years	Characteristics
Period 1	1980–1999	EBF for 4–6 months promoted for all mothers
Period 2	2000–2007	EBF 6 months promoted for HIV negative mothers; EBF for 3–6 months promoted or exclusive formula feeding (EFF) (if AFASS* met) for 6 months for HIV positive mothers
Period 3	2008–2011	EBF for 6 months promoted for HIV negative mothers EBF or EFF for 6 months promoted HIV positive mothers
Period 4	2012–2018	EBF for 6 months promoted for all mothers

*AFASS = Acceptable, Feasible, Affordable, Sustainable and Safe

As summarized in Table 1, while all mothers received the same advice during Period 1 and Period 4, between 2000 and 2011 different advice was provided to HIV positive and HIV uninfected mothers. Differences in advice went as far as indicating different durations of breastfeeding by HIV status and content (breastmilk or formula). For instance, during Period 2, HIV-positive mothers were officially counselled to either EBF for 3–6 months or EFF for 6 months before rapidly weaning [9], while HIV uninfected mothers were instructed to EBF for 6 months. South Africa’s prevention of mother to child transmission (PMTCT) guidance was amended in 2008 to encourage HIV-positive mothers to EBF for 6 months [8] in alignment with the country’s 2007 IYCF Policy [16]. Still, HIV positive mothers continued to be counselled that EFF for the first six months was an optimal option if they met AFASS criteria. Period 4 began in 2012, after South Africa declared itself a country that actively promoted, protected and supported EBF for all [10]. Free formula at public clinics was discontinued, with the exception of approved medical conditions. Currently, all health workers are expected to promote EBF for six months, regardless of the mother’s HIV status. If mothers decide to formula feed infants after counselling and they meet AFASS requirements, health workers can support them.

The overarching research question that the review sought to answer was ‘What factors support EBF for six months postpartum in South Africa?’ In order to inform policy and interventions, the review sought to explore EBF rates over time, and similarities or differences in the profiles or contexts of women who managed to exclusively breastfeed.

## Methods

### Protocol registration

The protocol first was registered with PROSPERO (International Prospective Register of Ongoing Systematic Reviews; http://www.crd.york.ac.uk/PROSPERO/ [registration number CRD42014010512). The Preferred Reporting Items for Systematic Reviews and Meta-Analyses (PRISMA) was used to guide the review [17].

### PICO (Population, Intervention, Comparator and Outcome) criteria

The key population of interest was mothers in South Africa. To be eligible, the mother had to have given birth to a healthy baby with known infant feeding practices through the first month postpartum. The intervention and/or exposures (referred to hereafter as factors) were left open intentionally, as these were the object of exploration. Significant factors and themes were noted from both observational studies and intervention evaluations, e.g. randomized controlled trials (RCTs) and quasi-experimental designs. The key comparator of interest was EBF. Overall, we looked at any breastfeed (yes vs. no) and, among those breastfeeding, the duration (point at which EBF was measured, e.g. 3 months vs. 6 months). For those studies that had the data, we disaggregated by the mother’s HIV status. The key outcome of interest was EBF for up to six months postpartum.

### Inclusion and exclusion criteria

To be included in the review, a number of criteria had to be met, as summarized in Table 2.

**Table 2:** Review selection criteria.

Selection Criteria	Standard
Study type	Peer-reviewed primary studies employing quantitative and/or qualitative methods as well as primary studies reported in the grey literature. Commentaries and opinion pieces will not be included. Studies where informed consent was not obtained were also excluded.
Languages	English, Spanish and French
Settings	South Africa. Multi-country studies will be permitted, but only data from South Africa will be extracted.
Publishing date	1980 to 2018 (data collection dates not older than 1975)
Outcomes	Breastfeeding practices (not intention to breastfeed)

### Search strategy

Seven databases were consulted through the University of the Witwatersrand (Wits) library: Africa-Wide, PubMed, Popline, PsychINFO, CINAHL, Global Health, and The Cochrane Library. Search strategies for each of the search engines were developed in consultation with two experts; the full search strategies can be found in **S1 File**. The search terms included variations of the following: infant feeding, breast feeding, bottle feeding, mixed feeding, solid feeding and South Africa, restricted for the period 1980 to 2018. The citations of relevant articles were also searched and content experts, e.g. UNICEF and South African Department of Health, were consulted to ensure all relevant articles were included.

### Selection and data extraction

The database searches were replicated independently by two of the authors (SJN and CBN) to reduce bias. The search results were independently saved as EndNote libraries and merged into a single library (n = 20,217) to remove duplicates; an additional 7 documents identified through consultations with experts, of which only one met eligibility criteria [18]. After removing duplicates (n = 2,528), SJN excluded 16,966 articles using titles and a further 293 using abstracts based on the selection criteria. SJN and CBN conducted full text screening on the remaining 101 eligible articles to arrive at the final list of 72 articles. All authors were consulted on the final list before data extraction was completed.

As a mixed-methods systematic review, qualitative and quantitative data were extracted and analyzed separately. All studies were uploaded into NVivo 10.0 software for systematic data extraction and analysis. Summary data for quantitative studies were extracted by both CBN and SJN into Microsoft Excel and SJN extracted qualitative study data. Both authors undertook random quality checks for each other. For both methods, we extracted information summarizing the study year, study design, study setting, sample, and infant age (signifying feeding duration). The policy period during which data were collected were also noted. This was based on separate identification of policy changes over time by SJN, already described in the introduction.

Some data extraction was specific to the methodology. For instance, analysis techniques were only noted for qualitative studies. A framework analysis approach [19] was applied to guide meta-synthesis of qualitative themes, which began by using the three socio-ecological levels and quantitative finding framings to categorize themes, only thereafter reporting on novel themes that were not included in the quantitative studies. The rationale for this approach was to facilitate a broader synthesis of findings from the two methodologies. For quantitative studies, two forms of summary measures were extracted. The primary descriptive measure for all studies was the EBF rate, which was presented as a percentage with notation for the duration that breastfeeding was measured. For the sub-set of intervention studies, the strongest analytic summary measure was extracted, most often in the form of odds ratios or hazard ratios. These were presented with confidence intervals (or p-values).

### Methodological quality and level of evidence assessment

The quality of studies was critically assessed using a range of tools: Grading of Recommendations Assessment, Development and Evaluation (GRADE) for RCTs [20–22], the Newcastle-Ottawa Scale (NOS) [23] for cohort studies, and an adaptation of NOS for observational studies. These were summarized as high, medium or low for the sake of comparison in the results summary table. CERQual [24, 25] was applied to critically assess qualitative studies. Although some qualitative researchers have argued against applying quality criteria for meta-synthesis or meta-ethnography, as it counters epistemological values of relativism, we adopted the position that basic standards of qualitative rigor (transferability, credibility, dependability and confirmability) should be present before the application of meta-synthesis [26]. For mixed methods studies, the appropriate tools already listed were applied to the different methodology and design. See **S2 File** for a full description of critical assessment methods and the assessments of each article.

## Results

**Fig 2:**
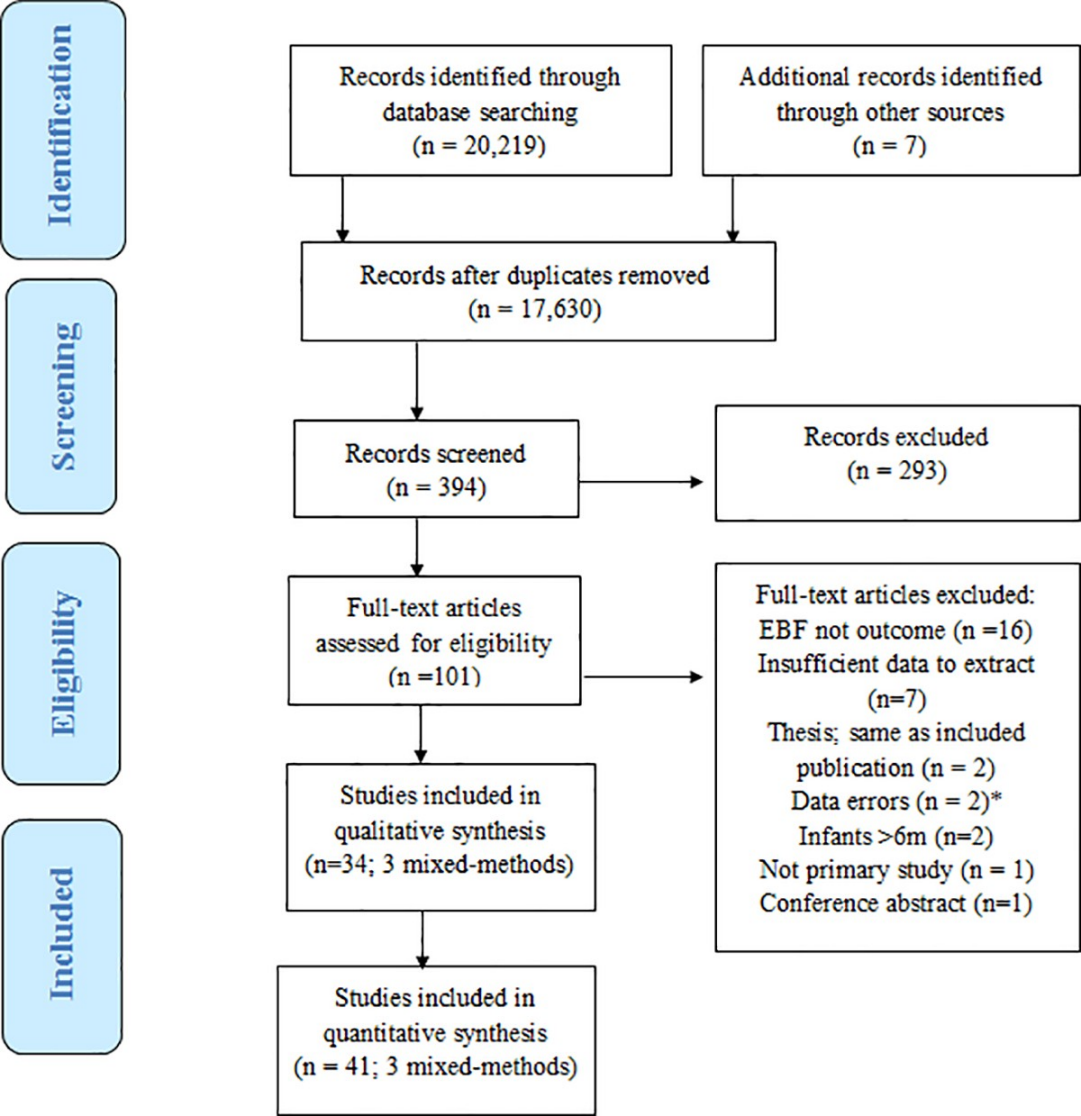
PRISMA flow chart on EBF supports in the first 6 months postpartum.

A total of 72 unique articles were reviewed; as three mixed-methods studies contributed to both sets of analyses, 41 articles were included in quantitative analysis and 34 articles were included in qualitative analysis (see Fig 2).

### Observations on excluded articles

As shown in Fig 2, articles were excluded mostly because EBF was not clearly defined as an outcome (n = 16) [27–43]or because the data were not presented in a format that aligned with preset review criteria (n = 7) [44–50]. This was particularly true for articles written in Periods 1 and 2 [20–29], when EBF was less a feature of “optimal feeding” definitions. The data from the two masters theses [51, 52] which were excluded are still represented in the review [53–55]. Whenever possible we retained articles, even if we had to recalculate EBF rates to enable comparison, e.g. when the denominator of EBF rates for HIV positive mothers excluded those who had opted to formula feed [11].

### Quantitative results

A total of 41 articles were identified using quantitative methods to explore EBF and one or more related determinants (see **S1 Table**).

### Quantitative studies characteristics

The quantitative studies on EBF in South Africa were highly varied in terms of both study design and quality. There was a relatively even spread of articles by policy period, demonstrating South Africa’s persistent scholarly interest in the topic of infant feeding. As detailed in **S1 Table**, while observational study designs dominated the mix (18 cross-sectional surveys, 10 cohort designs and one record review), there were also a number of intervention studies (four quasi-experimental designs, one trial and eight RCTs), with most RCTs in the last two policy periods.

Geographic biases were also clear in terms of where EBF was studied, clustering in Kwa-Zulu Natal (KZN; 17) [11, 18, 56–70], the Western Cape (WC; 13) [11, 53–55, 65, 71–78], and Gauteng provinces (GP; 10) [79–88]. Five studies reported data from the Eastern Cape (EC) [11, 65, 87, 89, 90]. The other provinces were poorly represented, with one study each in the Northwest (NW) [87], Mpumalanga (MP) [91] and Free State (FS) [87]. No studies were conducted in Limpopo (LP) or the Northern Cape (NC).

Many study sampling strategies introduced selection bias, likely to overestimate EBF (also see **S2 File**). For instance, in nine studies only mothers who had an intention to breastfeed or who were breastfeeding were included [54, 55, 65, 68, 73, 76, 84, 88, 89]; in these studies, exclusivity or introduction of complementary feeding were the main objects of interest. This was particularly true in research with HIV positive mothers during Policy Periods 2 and 3, as EFF was considered an acceptable alternative to breastfeeding [11, 18, 56, 57, 59, 61, 72, 89, 91]. Nearly all studies introduced selection bias by recruiting mothers from health facilities, often from prevention of mother to child transmission (PMTCT) programs or baby-friendly institutions, versus the community [62, 64, 66, 74]. The samples themselves also varied, though most involved mothers of young infants. In analysis, mothers were disaggregated by age, HIV status, race and/or in relationship to their exposure to an intervention. In a few cases, infant caregivers (versus mothers only) were sampled [53, 58, 87].

Most studies were scored as low to moderate quality (see **S2 File**). The introduction of bias in sampling was the most frequent reason for low or moderate scores. Other sources of weakness included small sample sizes, not powered to pick up differences, and limited use of advanced analytic techniques, such as regression analysis, to address confounding. Reliance on simple descriptive statistics or measures of association was the norm in studies conducted in the 1980s and 1990s, as compared with more recent studies. In addition, earlier studies were more likely to exclude details of the study, such as the relationship of the researchers to the sample and blinding for RCTs.

### EBF rates over time

There was a high level of hereogeneity in EBF durations reported (see **S1 Table**). Many early studies restricted measure of EBF to 12–16 weeks or less [58, 60, 61, 83], reflecting sampling strategies and definitions of optimal feeding at the time. The rates of EBF also varied widely by study sample and setting, even when we took duration into account. The articles presented EBF measures for different durations and used different definitions, such as including or excluding the use of non-prescribed medicine [58]. Only the strongest and longest duration measures of EBF are presented in Table 3 in order to assess EBF more critically. We summarized EBF prevalence ranges by policy period and study design (see Table 3, based on **S1 Table**).

**Table 3:** EBF ranges by policy period and study design.

Policy Period	EBF Ranges (Design)	Infant age at measurement
**1. 1980–1999**	0.0% to 32.0% (Cross-sectional)	6 months
	0.8%; 3.0% to 23.7% (Cohort)	6 months; 12–16 weeks
	32.7% to 38.7% (Quasi-experimental)	12 weeks
**2. 2000–2007**	0.0% to 5.0% (Cross-sectional)	6 months
	10.8% to 13%; *14*.*0% to 18*.*0%* (Cohort)	6 months; *14 weeks*
	45.0% (Quasi-experimental)	6 months
**3. 2008–2011**	*6*.*0% to 35*.*6%* (Cross-sectional)	*<6 months*
	1.5% to 6.4%; *20*.*1% to 20*.*8%* (RCTs)	6 months; *12 weeks*
**4. 2012–2018**	12.0%; *14*.*0%* (Cross-sectional)	6 months; *14 weeks*
	13.0% (Cohort)	6 months
	21.6% to 43.7% (RCTs)	6 months

While there was an upward trend in EBF over time, the rates varied widely within and across policy periods. For instance, during the first period, EBF for six months rates ranged from 0.0–32.0%. Interventions to increase EBF during this period did not measure to six months because guidelines at the time recommended 4–6 months. The next policy period, when PMTCT programs began, reported lower rates of EBF for six months (0.0–13.0%) across observational study designs, but a higher rate for the one quasi-experimental study [57]. Rates declined further during the third policy period in studies that measured EBF up to 6 months (1.5–6.4%). In policy period 4, after the Tshwane Declaration, EBF increased between 12.0–43.7%, with particularly high rates measured for RCTs specifically seeking to improve EBF for six months [68, 76].

### Quantitative findings on factors associated with EBF

A range of factors across individual, settings and structural levels were explored in relation to their influence on EBF. The factors identified in observational studies (cross-sectional, cohort and record reviews), significantly associated with EBF either as facilitators or barriers, were systematically documented according to the conceptual framework (see **S2 Table**). Where the findings were mixed, both barrier and facilitator symbols were used. Where regression analyses were not conducted with EBF as the outcome, only descriptive statistics could be used to characterize the study findings for EBF. In a few instances, secondary analyses from RCTs were integrated into this table.

The factors influencing EBF represented all three levels of the socio-ecological framework, with most related to mother attributes, hospital and family settings, and socio-cultural factors at the structural level. As quantitative studies can only report results on what they measure, the number of studies finding significant results for any given variable should be read with caution. For instance, only one study measured depression among mothers [69], but it found significant associations.

The quality of the studies and persistence of factors over time are both worth considering. A general trend in study quality was observed with earlier studies being less sophisticated, particularly with analysis, than those published in the last two policy periods. Some variable outcomes changed over time. For instance, while fear of HIV transmission used to be a barrier to EBF, more recently it has become a facilitator, with increased access to ART. This will be discussed further with the triangulation of quantitative and qualitative findings.

### Quantitative intervention results

Findings related to interventions are presented in Table 4, with final regression model results presented where multiple models were tested.

**Table 4:** EBF promotion intervention results.

Article and study design	Intervention description	Primary outcome	Results
**Policy period 1**			
Hoffman et al, 1984b [55] Pre-post, quasi-experimental	The intervention comprised six steps: 1. The birth notification form was duplicated so that one copy could be sent to the appropriate health visitor immediately after a birth while the other copy could be processed through the normal channels. 2. Breastfeeding clinics were introduced, allowing prospective mothers to see breastfeeding in action and learn by example. 3. Community talks on the advantages of breastfeeding. 4. Cape Town Breastfeeding Association contact numbers of volunteers available to help mothers with problems were circulated to all clinics in the area. 5. A letter to all the local family practitioners, encouraging breastfeeding promotion 6. Health visitors and nurses at the local clinic encouraged to promote breastfeeding. Their subject knowledge enhanced by lectures, demonstrations and a symposium.	‘Fully breastfed’	Significant findings observed **p<0.01 (chi-square test)** Pre-intervention <6w: 52.7% Post-intervention <6w: 75.8% **p<0.01 (chi-square test)** Pre-intervention 6–12w: 23.7% Post-intervention 6–12w: 38.7%
Nikodem et al, 1993 [83] Randomised control trial	Breastfeeding mothers were allocated, by means of randomly ordered cards in sealed opaque envelopes, to view one of two health education video programmes within 72 hours after delivery. The first programme gave information and specific motivation concerning the importance of breastfeeding and the correct positioning of the baby. The second gave information about healthy eating habits for adults. Results were tested 6 weeks postpartum through a blinded questionnaire interview, with 47.6% follow up rate.	‘Breastfed only’ at 6 weeks	Not significant for EBF at 6 w **OR 1.38 (.68–2.81); p = 0.431** Study: 30/83 (36.1%) Control: 23/79 (29.1%)
**Policy period 2**			
Baek et al., 2007 [18] Pre-post, quasi-experimental study design	Three evaluation sites in the Pietermaritzburg area of KZN recruited urban and peri-urban mothers (18–49) who knew their HIV status and were either 6–9 months pregnant or 12 weeks or less postpartum. Two cross-sectional surveys were conducted, baseline prior to mothers to mothers (m2m) intervention and another one year after m2m was introduced. At baseline data collection in 2005 before m2m was introduced. m2m was a peer support program that provided education and psychosocial support to HIV-positive pregnant women and new mothers through health talks, counselling & support groups and outreach. Two or more contacts were counted as study exposure.	Exclusive feeding practices: EBF	Not significant for EBF **p>0.05 (bivariate) for EBF** Study: 11% Control: 15%
		EFF	**p<0.01 (bivariate) for EFF** Study: 78% Control: 61%
Bland et al, 2008 [56] Non-randomized intervention cohort	Lay counsellors trained on the WHO/UNICEF Breastfeeding Counselling Course visited HIV-positive and HIV-negative women in KZN to support EBF: four times antenatally and once within 72 hours of birth. Mothers initiating breast- feeding received a further three home visits in the first 2 weeks and fortnightly thereafter for 6 months.	EBF* for six months *WHO definition	Significant for EBF at 4 months **aOR 2.07 (1.56–2.74), P<0.0001** HIV- mothers receiving visits **aOR 2.86 (2.13–3.83), P<0.0001** HIV+ mothers receiving visits
**Policy period 3**			
Ijumba et al, 2015 [62] Cluster-randomized trial	The intervention was provision of community-based counselling during the first 12 weeks after birth. The intervention was delivered by 15 trained CHW living in the clusters though a structured home visiting schedule. Each visit was designated to cover specific topics related to the outcomes of the study. Visits in the intervention arm included two home visits during pregnancy, one in the first 48 h after delivery, then at 3–4 d, 10–14 d, 3–4 weeks and a final visit at 8–9 weeks. All neonates with low birth weight (≤2500 g) received two extra visits during the first week.	EBF (24h recall) for the first 12 weeks *Adjusted for cluster*, *household asset score level and maternal education level*	Significant for EBF at 12w **Total: aOR 2.31 (1.82–2.93)** Study: 441/1629 (27.1%) Control: 260/1865 (13.9%) **HIV-: aOR 2.70 (2.01–3.70)** **HIV+: aOR 1.70 (1.32–2.20)**
Rotheram-Borus et al., 2014 [74] Cluster-randomized trial	The intervention was a home visiting intervention by community-based workers (CBWs) trained in cognitive-behavioural strategies to address health risks (by the Philani MCH and Nutrition Programme), in addition to clinic care (the control). CBW home visitors were selected from community role models prior to training.	EBF for six months	Significant for EBF at 6m **OR = 3.59 (1.91–6.75); p<0.001** Study: 10.3% Control: 3.1%
Some et al, 2017 [89] Clinical trial (RCT)	The intervention was provision of infant prophylaxis in the breastfeeding period plus one week from day 7 to 50 weeks of age with either lopinavir/ritonavir or lamivudine in four countries. HIV-1 positive mothers enrolled in the RCT were not eligible for HAART due to CD4 counts >350 cells/mm3. Country-specific hazard ratios for shorter duration of EPBF were calculated for a number of variables.	Combined EBF and Predominant BF into one group (EPBF) * *PBF very low	Significant for shorter EPBF **aHR 3.0 (1.6–5.5) for lop/rit** aHR 1.4 (1.1–1.9) age 25–30 aHR 1.6 (1.2–2.1) married aHR 1.3 (1.0–1.6) employed aHR 1.6 (1.2–2.1) multiparous
Tomlinson et al, 2014 [64] Community-based cluster RCT	Goodstart was a structured home visiting intervention where study CHWs provided two pregnancy visits and five post-natal home visits in Umlazi, Durban, South Africa. CHWs were living in the mothers’ neighbourhoods and received a10-day training on PMTCT, Integrated Management of Childhood Illness, lactation counselling and newborn care guidelines. They were also trained on motivational interviewing techniques. Control CHWs provided information and support on accessing social welfare grants and conducted three home-based visits: one during pregnancy and two during weeks 4–6 and 10–12 post-delivery.	EBF for 12 weeks	Significant for EBF at 12 weeks **RR 1.92 (1.59–2.33); all** Study: 28.6% EBF Control: 14.9% EBF **RR 1.53 (1.22–1.94); HIV+** **RR 2.16 (1.71–2.73) ; HIV1**
Tylleskar et al, 2011 [65] Cluster RCT	In the PROMISE-EBF intervention group, peers living around the study areas were trained for one week. Study peers provided 1 antenatal breastfeeding visit and 4 post-delivery visits. Control peer cousellors followed the same schedule but assisted families in obtaining birth certificates and social welfare grants. The peer counsellors for the intervention and control clusters were kept separate during the study.	EBF at 12 and 24 weeks using 24h and 7 days recall measures *Adjusted for clustering and site*	Significant for all EBF measures; 24 weeks prevalence ratios (PR) shown **PR 5.70 (1.33–24.26); 24 hr** **PR 9.83 (1.40–69.14); 7 day** Study: 2% (both 24h & 7 day) Control: <1% (both 24h & 7 day)
**Policy Period 4**			
Horwood et al, 2017 [66] Cluster RCT	The continuous quality improvement (CQI) intervention, CHWs provided home-based education and support to pregnant women and mothers. All CHWs received a10-day government training on community-based care of women and infants. Intervention CHWs received a 2-week training in WHO Community Case Management followed by 12 months of mentoring.	EBF for 6 weeks	Significant for EBF at 6 weeks **OR 1.7 (1.1–2.7); at follow-up** Study: 76.7% Control: 65.1% **OR 2.3 (1.4–4.0); change before and after intervention**
Myer et al, 2018 [76] Parallel arm RCT	The MCH-ART intervention provided integrated postnatal service to HIV+ mothers and their infants within the MCH clinic. At each postnatal visit nurse-midwives asked questions about infant feeding. The local standard of care acted as a control and involved immediate postnatal referral of HIV+ women on ART to general adult ART services and their infants to separate routine infant follow-up.	EBF at 6 months	Significant for EBF 6 months **p<0.001** Study: 67/211 (31.8%) Control: 26/219 (11.9%)
Reimers et al, 2017 [68] Cluster RCT	For this intervention, HIV+ mothers identified “Feeding Buddies” (FB) to support them. Two hour-long training ses- sions were scheduled prior to delivery at regular ANC visits. Two follow-up training sessions occurred at the 3 day and 6 week well-baby clinic visits. Where possible, the mother and her buddy were trained together. Mother-buddies were also given a take-home booklet to reinforce messages.	EBF at 22 weeks	Not significant for EBF at 22 w **p = 0.67** Study: 109/255 (42.75%) Control: 105/235 (44.68%)
Tuthill et al, 2017 [70] RCT	The Information–Motivation–Behavioural Skills (IMB) model was applied for HIV+ women on ART during their third trimester of pregnancy. The intervention was a one-time, 45-minute tailored, one-on-one motivational interviewing counselling session with a trained female counsellor. The control was standard of care.	EBF at 6 weeks	Not significant for EBF at 6 w **p = 1.00** Study: 81.5% Control: 81.5%

While most interventions showed statistically significant improvements in EBF (9/13), the variability of approaches made any prospect of meta-analysis impossible. As shown in Table 4, four studies did not observe significant differences between intervention and control groups. Short once-off interventions, such as playing a video [83] or a one-time counselling session [70], failed outright. Postnatal support by “peers” [18] or individuals nominated by mothers, “buddies” [68], had less success than interventions that used already-employed community health workers (CHWs) [56, 62, 64, 66]. The two RCTs that employed peer workers [65, 74] reported lower EBF rates than others. In other words, while their findings were statistically significant, the population-level benefit of increasing EBF to the levels they reported is questionable. Postnatal support provided by CHWs with at least 10 days training and between four to seven visits reported the most impressive EBF increases [64]. RCTs that applied health system reforms, such as integrated care for mothers and infants [76] or educating health staff [55], also resulted in significant EBF improvements.

### Qualitative findings

A total of 34 qualitative studies (including nine mixed-methods studies from which qualitative data were extracted), representing 31 distinct studies, were reviewed covering all policy periods (see **S5 Table)**. Four studies were conducted in the first period [92–95], three led by the sociologist Gill Seidel in KZN. Most studies were conducted during Period 2 (n = 14) [96–108] and Period 3 (n = 11) [53, 63, 109–117], highlighting the increased value placed on qualitative inquiry. In Period 4, five studies using qualitative methods were published on the influences on infant feeding in South Africa [118–122].

### Qualitative study characteristics

As with the quantitative studies, the range of qualitative study designs and settings was broad. Specific qualitative study designs included ethnography [96, 97, 107] and phenomenology [116], although 17/25 publications did not name a design or simply referred to their methods as qualitative. In terms of settings, studies were conducted in urban, peri-urban/township, and rural settings, with a bias for peri-urban and township environments. Mirroring the quantitative biases, the most popular study provinces were KZN (n = 13), WC (n = 13), GP (n = 8), and the EC (n = 6) followed by LP (n = 3) and the NW (n = 1) (see **S3 Table** for details). No qualitative studies meeting search criteria were published from Mpumalanga, Free State, or the Northern Cape provinces.

The people sampled for qualitative studies were strongly biased towards exploring the feeding influences of HIV positive mothers (23/34). Only 12 studies included mothers who were HIV negative or had an unspecified HIV status. Among mothers, some were purposively sampled based on age, parity, work status or feeding preference. The ages of the infants were not stated in all cases, and there was considerable variation when they were specified: neonates [110], mothers whose infants had an average age older than 6 months [99]; some whose infants had died [95]. This is relevant in terms of recall bias and how mothers may have described feeding influences. Health workers, particularly counsellors, were included in nine qualitative studies [53, 93, 94, 96–99, 108, 111]. Five studies included family members, usually fathers or grandmothers [53, 96, 97, 111, 112]. Some studies also included PTMCT or intervention program staff in their samples.

A total of 21 (61.8%) studies used interviews for data collection, with group discussions being the second most popular form of data collection (15/34), often with a mixture of the two. Novel [93–95] and participatory [108] methods were used in a few instances. Qualitative data were analyzed in a number of ways, with variations of thematic, content, and framework analyses the most common.

A meta-synthesis of themes from the qualitative studies was conducted by socio-ecological level prior to synthesis of the two methods (see **S5 Table**). The HIV epidemic and prevention efforts emerged strongly as an important part of the breastfeeding narrative. Following HIV positive mother narratives over time helped us understand their breastfeeding decision-making. Specifically, they helped us gain insight into how, as guidance about transmission risk changed from EFF to EBF, mothers’ behaviors also changed to provide the best health prospects for their infants. Gender and mixed feeding norms also emerged as strong infant feeding influences over time. To avoid repetition, these themes are synthesized below with the quantitative findings in order to highlight how the combined sources provide a more context-rich picture of infant feeding in South Africa.

### Synthesis of qualitative and quantitative findings across ecological levels

The synthesis of quantitative and qualitative study findings is presented in Table 5, with only statistically significant quantitative results or strong qualitative themes linked to EBF reported. The two unique features of this table are that, firstly, it accounts for variations in influences across time, and secondly these influences are described in relation to what the evidence found in relation to support for or against EBF. Where the data for influences were unclear, supporting EBF in some instances while acting as barriers in other studies, this is also noted.

**Table 5:** Synthesis of key influences on EBF from 1980–2018 (based on S2 and S4 Tables).

EBF influences by ecological level	Policy Periods	Evidence synthesis in relation to support (or not) for EBF
**STRUCTURAL–Sociocultural/ Market**		
Breastfeeding norms	All	Consistently supported EBF, with promotion project-based
Breastfeeding promotion	1–2 only	
HIV stigma against formula	All	Supports breastfeeding, but not exclusivity (see next influence)
HIV stigma around exclusive feeding	All	Undermins EBF; Exclusivity perceived as proxy for HIV
Mixed feeding norms before six months	All	Strong influence undermining EBF, with formula culture reinforcing norm
Commercial formula “culture”	3–4 only	
Social media/Internet	3–4 only	Reinforces existing biases/practices
Motherhood expectations and exclusive feeding	All	Sometimes, but not always, associated with “good” motherhood
**SETTINGS–Healthcare**		
Postnatal visits/support by HWs	All	Proactive visits strongly support EBF
Health worker counselling/advice	All	Strong influence; support of EBF depends on consistency and content
Separating mothers and infants	All	Consistently undermine EBF
Pre-lacteal feeds	1–2 only	
Free formula program	1–2 only	
**SETTINGS–Household**		
Support for mother after HIV disclosure	All	Supports EBF; for HIV-positive only
Family advice & caregiving support	All	Strong influence; EBF support depends on family preferences
Gender & power relations	All	Consistently undermine EBF, with rituals specific to only some cultures
Infant cleansing rituals	All	
**SETTINGS–Community**		
Community-based EBF support efforts	All	Strong influence; linked to projects
HIV stigma/gossip	All	Consistently undermine EBF; stigma fears strong for HIV-positive mothers
Work/school environments	All	
**INDIVIDUAL—Infant Attributes**		
Infant Growth	All	Healthy growth and calm disposition reinforce selected feeding practices
Disposition (crying, calm, etc.)	All	
Negative health events, e.g. HIV conversion	1–2 only	Negative infant responses undermine EBF
Breastmilk refusal	3–4 only	
**INDIVIDUAL—Mother attributes**		
Self-efficacy/confidence	2–4 only	Support EBF consistently
Knowledge of breastfeeding benefits	All	
Fear of HIV transmission (for HIV positive mothers only)	All	Strong influence for/against EBF; dependent on advice received
Past feeding experience	All	Strong influence for/against EBF; dependent on experience
Milk contamination beliefs	1–2 only	Consistently undermine EBF; milk contamination beliefs include HIV and other factors, such as not feeding breastmilk after sexual dreams
Antenatal depression	4 only	
Milk insufficiency beliefs	All	
Employed or in school	All	
Young and dependent on family	All	

Table 5 shows how most multi-level EBF influences persisted across all policy periods. Common influences that supported EBF were norms encouraging breastfeeding, postnatal support (from healthcare settings, community and households) and knowledge of breastfeeding benefits. Common EBF barriers included mixed feeding norms, the separation of mothers and infants after delivery, unsupportive workplaces (and schools), and milk insufficiency beliefs. The changes in influence that occurred between policy periods were concentrated in the healthcare setting and structural levels. In the healthcare setting we observed a move towards more breastfeeding-friendly environments, e.g. reducing pre-lacteal feeds and removing free formula. In contrast, the past decade has seen formula emerge as a “culture” and social media becoming increasingly important, coinciding with increased reports of infants refusing breastmilk.

True to our conceptual framework, some factors interacted across multiple levels of influence. For instance, HIV was seen with stigma at the sociocultural level, in how health workers advised mothers in the healthcare setting (often in conflicting ways), and through familial support (or not) after disclosure. At the individual level HIV manifested in how knowledge about breastfeeding benefits interacted with knowledge about transmission risks; these were dynamic. For instance, the fear of HIV transmission informed the choices of most HIV-positive mothers consistently, while the advice they received on the topic changed based on shifting PMTCT guidelines. Mothers with a recent HIV diagnosis appeared to be more influenced by infant feeding recommendations by health workers (who in Period 4 are more likely to promote EBF) than those with an older diagnosis.

Living in HIV endemic communities has influenced intentions to breastfeed and public performances of breastfeeding, even among non-infected women. For HIV-positive mothers, intentions to follow health advice were mitigated by fears that their HIV status would be disclosed inadvertently by their feeding practices; this was particularly clear in the qualitative studies [97, 100, 104, 112]. While active disclosure to intimate partners and family members was often described as a positive experience, some HIV-positive mothers avoided disclosure for fear of violence or abandonment [98]. When the government provided formula (Period 2), mothers did not want to be seen with the government brand of formula, Pelargon, and at times sold it to buy other brands or transferred the formula into empty tins of commercial brands [99, 104, 113]. Even when the free formula program ended, the practice of exclusive feeding (formula or breastmilk) was described with trepidation because avoidance of mixed feeding was perceived by community members to be linked to HIV [112], to the extent that HIV negative mothers report pretending to mix feed to avoid being labelled as HIV positive.

Other factors also operated across levels. Specifically, young and unemployed mothers were particularly vulnerable to abandoning EBF for reasons of gendered cultural expectations in their households and low perceived power. Those who wished to return to school opted for formula; expressing milk was not mentioned as a viable option, nor did any studies mention negotiating breastfeeding in the school setting. It was common for such young mothers to pass on child rearing responsibilities to their mothers; in doing so, they surrendered any ability to influence and determine decisions over infant feeding [112]. Within patriarchal kinship networks, young women faced expectations of unquestioning submission to the instructions of their elders, with mother-in-laws wielding particular power (if married). When elders differed in their beliefs about feeding, young mothers described deferring to family wishes and lying to health workers as their most common strategy to negotiate infant feeding. Financial dependence created added pressure to adhere to family wishes. In some instances, young mothers wanted to keep the baby’s father involved by requesting that he provide formula. Concerns about their body, e.g. saggy breasts, were also mentioned as barriers by younger women, although these concerns were mentioned less often than feelings of powerlessness in the family unit.

Older mothers faced their own challenges. Those who were employed or looking for work opted for formula or mixed feeding. Those with more than one child often based their decisions on how the firstborn fared. If a previous child survived mixed feeding, shifting to exclusive feeding was less likely, especially because of community mixed feeding norms.

Practical considerations were often raised. Mothers who felt that breastmilk was cheap and easy were most likely to breastfeed, whereas those who experienced pain and did not mind preparing formula went the opposite way. The discomfort and demands of breastfeeding, ranging from complaints of breast problems to sleepless nights, were rarely disclosed to new mothers; this lack of preparation was described as a reason for abandoning breastfeeding. These types of concerns have persisted over time.

As indicated, both young and older mothers reported family and community pressure to mixed feed across all four policy periods. Some of this advice comes from traditional practices or requirements. The most consistent reasons for complying with family pressure related to seeking to quiet a crying baby or fulfilling expectations about what an infant “needs.” Common beliefs about milk being insufficient also played a strong role at both household and community levels, reflecting and influencing mixed feeding norms. All of these factors contribute to whether a mother felt confident or had self-efficacy to EBF, which was strongly linked to the practice. One recent study also highlighted the role of mental health, particularly antenatal depression, as a barrier to EBF [69].

The healthcare setting has been noted as a critical space for establishing breastfeeding or not and as a source for breastfeeding (and HIV transmission) knowledge. Inconsistent and inadequate counselling by health workers was most often associated with abandoning EBF. Confused mothers often referred back to lay knowledge and direct observations of their children’s growth in a context where they were not given clear and consistent messages. This was most pronounced for HIV positive mothers. The free formula program, most often discussed during policy periods 2 and 3, eroded trust in EBF. While it no longer directly influences choice, confusion about optimal feeding among both mothers and health workers has carried into the current policy period. Other practices related to mother-baby friendly initiative (MBFI) hospitals were also reported across all periods. MBFI is South Africa’s localized version of the global Baby-Friendly Hospital Initiative (BFHI) [123]. While the practice of pre-lacteal feeds (health workers feeding infants formula before breastfeeding could be established) was only reported in the first two periods, experiences of mothers being separated from their infants after delivery persist. On the positive side, health services provided during the postnatal period, including home visits and support groups, were linked to increased and longer EBF, particularly when CHWs were involved. Our synthesis identified that neither school nor work settings promote EBF. Commercial formula marketing and the media space in general were understudied.

## Discussion

Our finding of highly variable rates of EBF over time was recently confirmed by spatial scientists as a trend both in South Africa and throughout the region [124]. While different policies and guidelines have influenced infant feeding, most obviously through health workers counselling practices and the free formula program for HIV positive mothers, this systematic review has highlighted persistent multi-level influences of EBF in South Africa over time for a larger population of mothers. The observation that complex factors constitute infant feeding decision-making [15] is clearly illustrated in this review.

At the structural level, norms that favor *both* breastfeeding *and* mixed feeding can partly explain high levels of breastfeeding initiation but early introduction of complementary feeding in South Africa [5]. Norms also influence gender identities, with one meta-ethnographic synthesis finding a narrative that breastfeeding was described as synonymous with “good mothering” [125]. The South African studies we reviewed suggest that this narrative is contested, as has also been found in the U.K. [126, 127]; for instance, some mothers who EBF based on health worker advice are chastised in the community setting for depriving their hungry infants of complementary foods [119]. Added to this, the mainstreaming of formula combined with the growth of social media present challenges to enforcing Code infringements [128].

The evidence suggests that the healthcare setting is moving towards more mother- and baby-friendly spaces, demonstrated in provincial plans [129]. The Tshwane Declaration has contributed to this shift. Our finding that both HW-led support and lay support can increase EBF compared to standard care has also been noted in two recent systematic reviews of the global literature, with health worker interventions showing the best results [130, 131]. While some factors influencing EBF require health systems responses, such as limiting the time mothers and infants are separated after delivery, many factors fall well outside of the health system’s purview, such as shifting norms.

Referring back to the adapted conceptual framework (see Fig 1) and synthesized results (see Table 5), a range of interventions are needed to address barriers to EBF and build on existing opportunities and efforts. These recommendations are made by level and draw on evidence from the reviewed articles, as well as, global literature.

Interventions at the individual level to support mothers and infants remain important as barriers related to milk insufficiency beliefs and the incompatibility of EBF with schooling and employment persist. Counselling and information sharing that clarifies the benefits of EBF for all infants [2] are needed for mothers, HWs as well as family members. For HIV positive mothers and the HWs treating them, the low risk of HIV transmission through breastfeeding while on ART needs emphasis [132]. Specific attention needs to be paid to how counsellors speak to all mothers about past feeding experiences, e.g. latching problems, and how the each infant is different. Ongoing efforts, such as a pediatric dietary guideline [133], are welcome in this context. Early diagnosis and treatment of depression among women postpartum is also likely to help [69]. For mothers facing HIV disclosure and the potential of violence or abandonment, specialized attention is needed. For young mothers, engagement with their families and school environments is most likely to increase EBF rates.

Within the community, norms around mixed-feeding, which is expected to be performed in public, must be addressed. The successful mass media and community mobilization campaigns by Alive and Thrive in Vietnam and Bangladesh are good models of how this might be done [134]. We agree with recommendations to address school and work policies and environments to shift perceptions that they are incompatible with breastfeeding [135, 136]. In South Africa, there is also clear scope to continue HIV stigma reduction efforts. This remains a huge barrier to HIV-positive mothers and a disincentive to EBF also for HIV-negative mothers, both in South Africa and in other HIV endemic settings.

The hospital setting presents a number of opportunities for institutions to align with MBFI practices, otherwise known as BFHI, particularly around keeping mothers and infants together after delivery. If this is not done, the practice of hospital staff separating mothers and infants will continue to undermine breastfeeding, as noted globally [4]. The importance of clear and compassionate counselling has been emphasized across all policy periods to promote (or discourage) EBF. This needs to include content clarity on duration and how to potentially overcome the challenges that may present early on.

Our synthesis highlights how families, particularly partners and surrogate caregivers, need to be engaged on how to support optimal feeding choices. They wield tremendous influence over infant feeding practices and by targeting these support structures, individual mothers could experience less pressure to negotiate exclusive feeding in a context where it is culturally not accepted. Strategies on how to acknowledge the source of socio-cultural beliefs and respect them, without endangering the infant’s health, are also needed. This process has already started, taking direction from the Tshwane Declaration and Regulation 991 [10, 137], and through campaigns such as Side by Side (sidebyside.co.za). These could be expanded.

A limitation of this review is that only one author did the initial screening of the full sample to identify potentially eligible articles, while pairs for the entire process are recommended [138]. Despite the large number of articles in the final review, significant population groups have not been studied. Almost all studies are with African women. Cultural considerations of different ethnic groups, including Indian populations (both Muslim and Hindu) and whites, are absent from these studies. Higher socio-economic status women have been largely neglected as well. Few studies have been conducted in four provinces of South Africa’s nine provinces. As mentioned earlier, there has also been a much stronger focus on mothers, leading to gaps in our understanding about work and school environments, and accommodation of infant feeding. The role of the media in promoting or undermining breastfeeding is also missing from conceptual frameworks. All of these gaps provide opportunities for future research. Many studies did not meet PICO requirements but still addressed many of the questions raised about EBF. Finally, the quality of studies was highly variable. We applied an exploratory approach to uncover as many factors as possible, rather than excluding factors based on poor study design, except in a minority of cases discussed earlier. However, the variability of designs meant that we were unable to conduct a meta-analysis.

## Conclusion

Exclusive breastfeeding for six months is possible, but it is challenging in the South African context. Multi-level barriers to EBF, highlighted globally [4], were described across all four policy periods. These included milk insufficiency beliefs, separating mothers and infants after delivery, and mother returning to work. In addition, South Africans contend with strong mixed feeding norms and an HIV epidemic that permeates all levels [139]. South Africa is also extremely diverse, with 11 official languages and myriad cultural permutations not reflected in our literature. This review highlights gaps in our understanding of infant feeding in various populations, both geographically and in terms of sociodemographic backgrounds. It also highlights potential blindspots in our focus on the market context where formula and breastfeeding are promoted (or not) and that mixed feeding norms require creative approaches that go beyond simply counselling individuals.

## Supporting information

S1 File Search strategies. (PDF)

S2 File Quality scoring standards and results. (PDF)

S1 Table Quantitative article summary table. (PDF)

S2 Table Quantitative results for observational studies. (PDF)

S3 Table Qualitative article summary table. (PDF)

S4 Table Qualitative meta-synthesis. (PDF)

S5 Table PRISMA checklist. (PDF)
