# Supplementary material for: Exclusive breastfeeding policy, practice and influences in South Africa, 1980 to 2018: A mixed-methods systematic review
Source: PLoS One. 2019 Oct 18;14(10):e0224029. doi: 10.1371/journal.pone.0224029 (PMC6799928; doi:10.1371/journal.pone.0224029)
Supplement: S1 File — (PDF) [file pone.0224029.s001.pdf]

S1 File. Systemic review search strategies

1&2. PubMed & PsychInfo*(with date restrictions from 1980)*
1. (infant feed* or (bottle adj3 feed*) or (breast adj3 (milk or fed or feed*)) or breastfeed* or breastfed or breast-feed* or breast-fed).ti,ab.
2. infant feeding/ or bottle feeding/ or breast feeding/ or solid feeding/
3. 2 or 3
4. South Africa/ or "South Africa*".ti,ab.
5. 3 and 4

3. Global Health (CAB)
1. South Africa/ or "South Africa*".ti,ab.
2. infant feeding/ or bottle feeding/ or breast feeding/ or solid feeding/ or infant formulae/
3. (infant feed* or (bottle adj3 feed*) or (breast adj3 (milk or fed or feed*)) or breastfeed* or breastfed or breast-feed* or breast-fed).ti,ab.
4. 2 or 3
5. 1 and 4
6. limit 5 to yr="1980 -Current"

4. Popline
All Fields: ((infant AND (formula OR fee*)) OR (bottle AND fee*) OR (breast AND (milk OR fed OR fee*)) OR breastfee* OR breastfed) **AND**
Region/Country: South Africa

5 & 6. Cinahl & Africa-Wide
S1 TI("South Africa*") OR AB("South Africa*") OR SU("South Africa*") Limiters - Published Date: 19800101-20140631 [Database - CINAHL Plus with Full Text]
S2 TI((infant N3 (formula or feed*)) or (bottle N3 feed*) or (breast N3 (milk or fed or feed*)) or breastfeed* or breastfed or breast-feed* or breast-fed) OR AB((infant N3 (formula or feed*)) or (bottle N3 feed*) or (breast N3 (milk or fed or feed*)) or breastfeed* or breastfed or breast-feed* or breast-fed) OR SU((infant N3(formula or feed*)) or (bottle N3 feed*) or (breast N3 (milk or fed or feed*)) or breastfeed* or breastfed or breast-feed* or breast-fed) [Database - CINAHL Plus with Full Text]
S3 S1 AND S2 [Database - CINAHL Plus with Full Text]

7. Central Register of Trials
1. ("infant feed" or "infant feeds" or "infant feeding" or (bottle near feed*) or (breast near (milk or fed or feed*)) or breastfeed* or breastfed or breast-feed* or breast-fed):ti,ab
2. [mh ^"infant feeding"] or [mh ^"bottle feeding"] or [mh ^"breast feeding"] or [mh ^"solid feeding"]
3. [mh ^"South Africa"] or ("South Africa" or "South African" or "South Africans" or "South Africa's"):ti,ab 4. (#1 or #2) and #3 – Limited by date 1980-Present
