# Supplementary material for: Exclusive breastfeeding policy, practice and influences in South Africa, 1980 to 2018: A mixed-methods systematic review
Source: PLoS One. 2019 Oct 18;14(10):e0224029. doi: 10.1371/journal.pone.0224029 (PMC6799928; doi:10.1371/journal.pone.0224029)
Supplement: S2 File — (PDF) [file pone.0224029.s002.pdf]

S2 File. Article quality scoring standards and results

Quality Assessments for Systematic Review
Adapted Newcastle-Ottawa Scale (NOS) for
Cross-sectional and Cohort studies
and GRADE for RCTs

We downloaded the following scale from: http://www.ohri.ca/programs/ clinical_ epidemiology /oxford.asp, to evaluate the quality of included studies. These were then adapted to better reflect nuances of study design.

1. Adapted Newcastle-Ottawa Quality (aNOS) Assessment Scale: Cross- Sectional Studies (Low=0-2; Medium=3-4; High=5-7)
Note: A study can be awarded a maximum of one star for each numbered item within the Selection and Exposure categories.

Selection
1) Is the outcome definition adequate? (max. 2)
   a) yes, with independent validation ⁕⁕
   b) yes, eg based on WHO measure or validated self report⁕
   b) yes, eg record linkage or based on non-validated self report
   c) no description
2) Representativeness of the sample (max. 2)
   a) consecutive or obviously representative series of cases ⁕⁕
   b) potential for selection biases or not stated
3) Selection of Sample (max. 1)
   a) community-based ⁕
   b) hospital-based
   c) no description

Exposure
1) Ascertainment of exposure (max. 1)
   a) secure record (eg surgical records) ⁕
   b) structured interview ⁕
   c) written self report or medical record only
   d) no description

Analysis
1) Analysis of findings (max. 2)
   a) Regression at 95% confidence ⁕⁕
   b) Bi-variate tests of associations ⁕
   c) Descriptive only

Table S2.1 Detailed Assessment of Cross-sectional Studies

	Selection 1) Outcome	Selection 2) Representative	Selection 3) Sample	Exposure	Analysis	Score
Chalmers, 1986	Self-report low	Not stated	Hospital	Questionnaire	Association	2 - Low
				✸	✸	✸✸
Chalmers, 1987	Self-report low	Not stated	Homes	Questionnaire	Descriptive	2 - Low
			✸	✸		✸✸
Delport et al, 1988	Self-report low	Selection bias	Hospital	Questionnaire	Descriptive	1 - Low
				✸		✸
Ellison et al, 1997	Self-report low	Not stated	Hospital	Questionnaire	Association	2 - Low
				✸	✸	✸✸
Hoffman et al, 1984a	Self-report low	Selection bias	Clinic	Questionnaire	Descriptive	1 - Low
				✸		✸
Hoffman et al, 1984a	Self-report low	Selection bias	Clinic	Questionnaire	Association	2 - Low
				✸	✸	✸✸
MacIntyre et al, 2005	Self-report	Selection bias	Hospital	Questionnaire	Association	3 - Medium
	✸			✸	✸	✸✸✸
Van der Elst et al, 1989	Self-report low	Selection bias	Hospital	Questionnaire	Association	2 - Low
				✸	✸	✸✸
Kassier et al, 2003	Self-report	Representative	Health centre	Questionnaire	Association	4 - Medium
	✸	✸		✸	✸	✸✸✸✸
Petrie et al, 2007	Self-report	Not stated	Health centre	Questionnaire	Association	1 - Low
				✸	*$p<0.1$*	✸
Sibeko et al, 2005	Self-report	Representative	Clinic	Questionnaire	Association	4 - Medium
	✸	✸		✸	✸	✸✸✸✸
Goosen et al, 2014	Self-report	Representative	Community	Questionnaire	Association	5 - High
	✸	✸	✸	✸	✸	✸✸✸✸✸
Ladzani et al, 2011	Self-report	Representative	Clinic	Questionnaire	Regression	5 - High
	✸	✸		✸	✸✸	✸✸✸✸✸
Swarts et al, 2010	Self-report	Sampling bias	Clinic	Questionnaire	Association	3 - Medium
	✸			✸	✸	✸✸✸
Mnyani et al, 2017	Self-report low	Representative	Clinic	Questionnaire	Regression	4 - Medium
		✸		✸	✸✸	✸✸✸✸
Pillay et al, 2018	Self-report low	Selection bias	Clinic	Questionnaire	Regression	3 - Medium
				✸	✸✸	✸✸✸
Siziba et al, 2016	Self-report	Representative	Health facilities	Questionnaire	Association	4 - Medium
	✸	✸		✸	✸	✸✸✸✸
Steyn et al., 2017	Self-report low	Selection bias	Private practices	Questionnaire	Association	2 - Low
				✸	✸	✸✸
Yako & Nzama, 2013	Self-report	Selection bias	Health centre	Questionnaire	Association	3 - Medium
	✸			✸	✸	✸✸✸
Du Plessis, 2009	Self-report	Selection bias	Private practice	Questionnaire	Descriptive	2 - Low
	✸			✸		✸✸

Baek et al, 2007	Self-report	Selection bias	Health facilities	Questionnaire	Regression	4 - Medium
	✸			✸	✸✸	✸✸✸✸

2. Adapted Newcastle-Ottawa Quality Assessment Scale: Cohort Studies

Note: The maximum number of stars is indicated for each numbered item within the Selection and Outcome categories. A maximum of two stars can be given for Comparability. Nine star maximum for non-interventions and 11 for interventions. Non-intervention cohorts: 0-3=Low, 4-6=Medium, 7-9=High; Intervention: 0-4=Low, 5-7=Medium, 8-11=High

Selection

1) Representativeness of the exposed cohort
   a) truly representative of the average _____(describe) in the community⁕⁕
   b) somewhat representative of the average ________in the community⁕
   c) selected group of users eg nurses, volunteers
   d) no description of the derivation of the cohort

2a) Selection of the sample for interventions
   a) control drawn from the same community as the exposed cohort ⁕
   b) control drawn from a different source
   c) no description of the derivation of the non-exposed cohort

2b) Selection of sample for descriptive
   a) community-based ⁕
   b) hospital-based
   c) no description

3) Ascertainment of outcome
   a) structured interview, either WHO or validated tool ⁕
   b) self report, (validation not reported)
   c) no description

4) Demonstration that outcome of interest was not present at start of study
   a) yes ⁕
   b) no

Comparability (intervention only)

1) Comparability of cohorts on the basis of the design or analysis (max 2)
   a) study controls for exposure to intervention⁕
   b) study controls for any additional factors, e.g. HIV status ⁕

Outcome

1a) Analysis of outcome
   a) independent blind assessment ⁕⁕
   b) record linkage ⁕
   c) self report
   d) no description

1b) Analysis of outcome (descriptive)
   a) Regression at 95% confidence ⁕⁕
   b) Bi-variate tests of associations ⁕
   c) Descriptive only

2) <u>Was follow-up long enough for *'meaningful'* outcomes to occur</u>

a) yes, 12+ weeks ⁕

b) yes, <12 weeks

c) no

3) <u>Adequacy of follow up of cohorts</u>

a) complete follow up - all subjects accounted for ⁕

b) subjects lost to follow up unlikely to introduce bias - small number lost - > 75% follow up, or description provided of those lost) ⁕

c) evaluation of subjects lost to follow indicate biases that are not controlled for in analysis

d) follow up rate < 75% and no description of those lost

e) no statement

Table S2.2 **Detailed Assessment of Cohort Studies**

	Selection Representa-Tive	Selection Sample	Selection Exposure	Selection Outcome-w/o	Comparability (intervention only)	Outcome Analysis	Outcome FU length	Outcome FU rate	Score
Bland et al, 2007	Biased	Hospital	Self-report	Yes	N/A	1b Reg.	1 week	Examined	Med
			✵	✵	-	✵✵		✵	5
Bork et al, 2013	Not stated	Hospital	Self-report	Yes	N/A	1b Reg.	>12 week	Biased	Med
			✵	✵	-	✵✵	✵		5
Bland et al., 2008	Biased	Hospital	Self-report	Yes	Visits + HIV	1a Blinded	>12 week	Not discussed	Med
			✵	✵	✵✵	✵✵	✵		7
Goga et al, 2012	Yes - consecutive	PMTCT sites	Self-report	Yes	N/A	1b. Assoc.	>12 week	Biased	Med
	✵		✵	✵	-	✵	✵		5
Ghuman et al, 2009	Not stated	Hospital	Unclear	Yes	N/A	1b. Assoc.	>12 week	Biased	Low
				✵	-	✵	✵		3
Budree et al, 2017	Not stated	Clinics	Self-report (FFQ)	Yes	N/A	1b Reg.	>12 week	Not discussed	Med
			✵	✵		✵✵	✵		5
Thomas et al., 2017	Not stated	Clinics	Self-report	Yes	N/A	1b Reg.	>12 week	Not discussed	Med
			✵	✵		✵✵	✵		5
Tuthill et al, 2017	Biased	Clinics	Self-report - low	Yes	N/A	1b Reg.	6 weeks	Not discussed	Low
				✵		✵✵			3
Sepeng & Ballot, 2016	Yes	Neonatal ward	Records	Yes	N/A	1b Reg.	<12 weeks	N/A	Med
	✵		✵	✵		✵✵			5

**Table S2.3** Detailed Assessment of RCTs using GRADE criteria											
**Study**	**Quality**						**Effect**				
	**Design**	**Risk of bias**	**Inconsistency**	**Indirectness**	**Imprecision**	**Other**	**Size - Trial**	**Size-Control**	**Relative Effect**	**Quality summary**	**Importance**
Nicodem et al,	RCT	Moderate	Low	Low	Moderate	Low	36.1%	29.1%	1.38 (ns)	Moderate	Important
	Video	Random allocation & blinding; only mothers who breastfeeding & follow-up low (47.6%)	All plausible	Clear links	No regression analysis		30/83	23/79	6 weeks		
Ijumba et al, 2015	Cluster RCT	Low	Low	Low	Low	Low			2.29	High	Critical
	CHW	Blinded, allocated	Sub-group analysis by HIV	Clear links	Clear measures		d-1629	d-1865	12 weeks		
Rotheram-Borus et al., 2014	Cluster RCT	Moderate	Low	Low	Low	Low	71.3%	52.1%	2.38	Moderate	Important
	EI vs. SC	Selection effects; No blinding; Not allocated	All plausible	Clear links	Clear measures		n-57 d-	n-50 d-	6 month		
Some et al, 2017	Clinical trial	Moderate	Low	Moderate	Moderate	Low	1.0	3.0	Missing	Low	Low
	ART types	Only mothers planning to breastfeed; blinding unclear	All plausible	ART regime & EBF links unclear	EPBF vs EBF measured		HR for lam. (short EPBR)	HR for Lop/rit (short EPBR)	1 week		
Tomlinson et al, 2014	Cluster RCT	Low	Low	Low	Low	Low	28.6%	14.9%	1.92	High	Critical
	CHWs	Blinding; random allocation; low attrition	Sub-group analysis by HIV	Clear links	Clear measures		430/ 1373	252/ 1693	12 weeks		

Tylleskar et al, 2011	Cluster RCT	Moderate	Low	Low	Low	Low	2.0%	<1.0%	5.70	Moderate	Important
	CHW	Only mothers intending to breastfeed; random allocation; mod attrition (>15%) in 1 arm	All plausible	Clear links	Clear measures		12/ 535	2/ 485	24 weeks		
Horwood et al, 2017	Cluster RCT	Low	Low	Low	Low	Low	76.7%	65.1%	1.7	High	Critical
	QI for CHWs	Random allocation; no blinding; mod attrition in both arms	All plausible	Clear links	Not WHO standards		194/ 253	181/ 278	6 weeks		
Myer et al, 2018	RCT	Moderate	Low	Low	Low	Low	31.8%	11.9%	Missing	Moderate	Important
	Integrated care	Mothers breastfeeding before 6w	Plausible & sub-groups tested	Clear links	Clear measures		67/ 211	26/ 219	6 months		
Reimers et al., 2017	Cluster RCT	Moderate	Low	Low	Moderate	Low	42.8	44.7	Missing	Moderate	Important
	Feeding Buddies (FB)	Mothers planning EBF; moderate attrition	All plausible	Clear links	FBs were not standard in terms of delivery		109/ 255	105/ 235			
Tuthill et al, 2017	RCT	Low	Low	Low	Moderate	Low	81.5%	81.5%	1.0	Moderate	Important
	Theory-based counseling vs. SOC	Blinding and random allocation; low attrition	All plausible	Clear links	Sample not powered adequately		N=29	N=29	N=58		
