# Supplementary material for: Exclusive breastfeeding policy, practice and influences in South Africa, 1980 to 2018: A mixed-methods systematic review
Source: PLoS One. 2019 Oct 18;14(10):e0224029. doi: 10.1371/journal.pone.0224029 (PMC6799928; doi:10.1371/journal.pone.0224029)
Supplement: S1 Table — (PDF) [file pone.0224029.s003.pdf]

S1 Table. Summary of quantitative studies, longest EBF rates and quality outcome

#	Article Ref	Design	Setting*	Sample Size	Groups Assessed	Infant Age(s)	EBF [ages]**	Quality (H/M/L)
**Period 1 (1980-1999)**								
1	Bland et al., 2002	Cohort<br><br>Cross-sectional	R (Hlabisa, KZN)	119 (cohort)<br><br>101 (x-sect)	Infant of mothers attending rural clinics (to 16w)<br>101 mothers or caregivers with infants below 6m	<16 weeks<br><br>17-24 weeks	3% (16w; n=119)<br><br>22.0% (6m; n=101)	High -aNOS
2	Chalmers, 1986	Cross-sectional	R & U (GP)	171	Pedi mothers who gave birth in a clinic or hospital	Not stated	69.0% (n/s; n=171)	Low -aNOS
3	Chalmers et al., 1987	Cross-sectional	U (Johannesburg, GP)	200	Coloured (mixed race) mothers who had given birth in a hospital	~3 weeks	82.0% (3w; n=200)	Low -aNOS
4	Delport et al, 1988	Cross-sectional	U (GP)	51	Breastfeeding mothers at private maternity hospital	4-5 days	43.1% (5d; n=51)	Low -aNOS
5	Ellison et al., 1997	Cohort	U/T (Soweto, GP)	1267	Mothers enrolled in Birth to 10 cohort, Soweto	<6 months	0.8% (6m; n=not reported)	Low -aNOS
6	Hoffman et al, 1984a	Cohort	U (Heideveld, WC)	169<br>*-80*	Mothers<br>*-80/89 mothers who reported EBF at 1st interview*	<6 weeks<br>7-12 weeks	23.7% (16w; n=80)	Low -aNOS
7	Hoffman et al, 1984b	Quasi-experimental (Pre and Post)	U (Heideveld, WC)	124 (Post)<br>*-94*	Mothers for post-intervention assessments were new, but compared with mothers from previous study (see 1984a)	<6 weeks &<br>7-12 weeks for both groups	38.7% (12w; n=44)	Low -aNOS
8	MacIntyre et al., 2005	Cross-sectional	U (Ga-Rankuwa, GP)	150	Mothers of healthy infants attending a clinic	<9 weeks	4.7% (<9w; n=150)	Medium -aNOS
9	Nikodem et	Randomised	U (Coronation	162	Low-income mothers who	<12 weeks	32.7%	Moderate

S1 Table. Summary of quantitative studies, longest EBF rates and quality outcome

	al, 1993	controlled trial	Hospital, GP)		gave birth at Coronation Hospital and participated in intervention study (83) or were controls (79)		(<12w; n=162)	-GRADE
10	Van der Elst et al, 1989	Cross-sectional	T (Mitchell's Plain, WC)	78	Mothers (Black & coloured) who had given birth at a baby-friendly maternity hospital at a 6-month follow-up home visit	6 months	32.0%<br>(6m; n=78)	Low<br>-aNOS
**Period 2 (2000-2007)**								
11	Baek et al., 2007	Quasi experimental (pre- post cross-sectional surveys)	U (Edendale hospital, KZN)<br>PU (Imbalenhule & Mpophonemi, KZN)	320	HIV-positive mothers who provided data on infant feeding practices and exposure to the m2m programme (2x or more).	<3 months	8.1%<br>(4-12w; n=320)	Medium<br>-aNOS
12	Bland et al, 2007	Cohort (pregnancy and postnatal)	R (KZN)<br>PU (KZN)<br>U (KZN)	2260<br><br>*-1156*<br>*-1104*	Mother/infant pairs with infant feeding data<br>*-HIV positive (863)*<br>*-HIV negative (902)*	1 week postnatal	78.1%<br>(1w; n=2260)<br>*74.7% HIV+*<br>*81.7% HIV-*	Medium<br>-aNOS
13	Bland et al, 2008	Quasi-experimental (cohort)	R (KZN)<br>PU (KZN)<br>U (KZN)	2436<br>*-1219*<br>*-1217*	Mothers<br>*-HIV positive*<br>*-HIV negative*	< 180 days (6 months)	45% HIV-<br>40% HIV+<br>(6m)	Medium<br>-aNOS
14	Bork et al, 2013	Cohort	U (Durban, KZN)<br>R/PU (Somkhele, KZN)	278	HIV-1 positive mothers with CD4 between 200-500	<6 months	10.8%<br>(6m; n=269)	Medium<br>-aNOS
15	Du Plessis, 2009	Mixed methods (for quant: 2 cross-sectional questionnaires)	U (Johannesburg, GP)	124<br>*-55 (phase 1)*<br>*-69 (phase 2)*	Primigravidae mothers who breastfed or attempted to breastfeed for at least 3 weeks who attended a baby clinic	>6 weeks	39.0%<br>(6w; n=124)	Low<br>-aNOS

S1 Table. Summary of quantitative studies, longest EBF rates and quality outcome

16	Goga et al, 2012	Cohort study	R (Rietvlei, EC)<br>PU/T (Paarl, WC, Umlazi, KZN)	775 (at 3 weeks)	Mothers were recruited during pregnancy with a HIV- positive & -negative enrolment (3:1)	<36 weeks (visits at 3, 5, 7, 9, 12, 16, 20, 24, 28, 32 and 36 wks)	14.0%<br>(12-14w; n=665)	Medium<br>-aNOS
17	Ghuman et al, 2009	Cohort study	R (Ugu, KZN)	168 (birth)<br>*-117 (14 weeks)*	Mothers who gave birth at baby friendly clinic	Birth<br>14 weeks	18.0%<br>(14w; n=117)	Low<br>-aNOS
18	Kassier et al, 2003	Cross-sectional	U (Durban, KZN)	300<br>*-150*<br>*-150*	Zulu mothers<br>*Attending PTMTC clinics*<br>*Attending non- PTMTC*	<6 weeks<br>6-14 weeks<br>15 weeks-6 m	5.0%<br>(15w-6m; n=80)	Medium<br>-aNOS
19	Petrie et al, 2007	Cross-sectional	U (Cape Town, WC)	20	HIV positive mothers age 18-39 attending community health clinic with infant feeding data	<6 months	5.0%<br>(<6m; n=20)	Low<br>-aNOS
20	Sibeko et al, 2005	Cross-sectional	PU/T (Langa, WC)	117	115 mothers with 117 healthy babies, currently breastfeeding	1-6 months	0.0%<br>(1-6m; n=117)	Medium<br>-aNOS
**Period 3 (2008-2011)**								
21	Goosen, McLachlan & Schübl, 2014	Mixed methods (Cross-sectional)	PU/T (Avian Park & Zwelenthemba, WC)	140	Primary caregivers of infants aged 0 to 5.9 months in study area	0-5.9 months	6.0%<br>(<6m; n=140)	High<br>-aNOS
22	Ijumba et al, 2015	Cluster-randomised trial	PU (Umlazi, KZN)	3494<br>*1629 study*<br>*1865 control*	Mothers from 30 community clusters at 12 weeks in GoodStart RCT	12 weeks	20.1%<br>(12w; n=3494)	High<br>-GRADE

S1 Table. Summary of quantitative studies, longest EBF rates and quality outcome

23	Ladzani et al, 2011	Cross-sectional	R (Gert Sibande, MP)	815	HIV positive mothers from 47 PMTCT clinics	3-6 months	35.6% (3-6m; n=815)	High -aNOS
24	Rotheram-Borus et al., 2014	Cluster randomised controlled trial	T (Cape Town, WC)	1238	Mothers from 24 neighbourhoods (12 study matched with 12 control)	<6 months (2 week & 6 month visits)	6.4% (6m; n=1157)	Moderate -GRADE
25	Some et al, 2017 *[Data collected 2009-2013; relevant data pre-2012]*	Clinical trial	U (East London, EC)	213	Adult (age 18+) HIV+ mothers attending ineligible for HAART who expressed intention to breastfeed at enrollment	<26 weeks	93.4% (1 w; n=213)	Low -GRADE
26	Swarts, Kruger & Dolman, 2010	Mixed methods (Cross-sectional)	R (Lower Umfolozi District, KZN)	100 *-37 HIV+* *-63 HIV-/unknown*	Mothers in post-natal ward of baby-friendly hospital *-HIV status retrieved from clinical records*	Neonates	72.0% (<1w; n=100)	Medium -aNOS
27	Tomlinson et al, 2014	Cluster randomised controlled trial	PU (Umlazi, KZN)	3494 *1629 study* *1865 control*	Mothers from 30 community-based clusters at 12 weeks in GoodStart RCT	12 weeks	20.8% (12w; n=3494) *24% study* *16% control*	High -GRADE
28	Tylleskar et al, 2011	Cluster randomised trial	R (Rietvlei, EC) PU/T (Paarl, WC, Umlazi, KZN)	1020 *535 study* *485 control*	Mothers who planned to breastfeed prior to giving birth	<28 weeks	1.5% (22-24w; n=1020)	Moderate -GRADE
**Period 4 (2012-2018)**								
29	Budree et al, 2017	Cohort	PU (Paarl, WC)	1076	Infants of 1071 mothers age 18+ in cohort of a mixed-race community and Black-African community	6 months (6-10, 14-16, and 6m)	13.0% (6m; n=710)	Medium -aNOS

S1 Table. Summary of quantitative studies, longest EBF rates and quality outcome

30	Horwood et al, 2017	Cluster randomised controlled trial	R (Ugu Health District, KZN)	1342 total<br>*736 baseline*<br>*606 follow-up*	Adult (age 18+) mothers with an infant <12 months and living in a household served by a participating CHW (both HIV+ and -)	6 weeks (for EBF measure)	68.8%<br>(6w; n=629 baseline)<br>70.6%<br>(6w; n=531 follow up)	High -GRADE
31	Myer et al, 2018(Myer et al., 2018)	Randomised controlled trial	U (Cape Town, WC)	471<br>*233 Study*<br>*238 Control*	Mothers (age 18+) HIV+ at MCH clinic less than 6 weeks postpartum who had initiated ART during pregnancy and were breastfeeding at screening	<6m<br>(3m and 6m)	21.6%<br>(6m; n=430)	Moderate -GRADE
32	Mnyani et al, 2016	Cross-sectional	PU (Soweto, GP)	180<br>*67 HIV+*<br>*113 HIV-*	Mothers accessing primary health clinics	<9 months (avg age 5.5 HIV- and 5.9 HIV+)	68.0%<br>(<6m; n=100)	Medium -aNOS
33	Pillay et al, 2018	Cross-sectional	R (Umlazi, KZN)	73	Teenage (<19) mothers attending a Well-Baby Clinic for 14-week immunization	14 weeks	50.7%<br>(14w; n=73)	Medium -aNOS
34	Reimers et al., 2017	Cluster randomised trial	U/PU/R (uThungulu District, KZN)	490<br>*255 study*<br>*235 control*	Adult (age 18+) HIV+ mothers attending PMTCT program with intention to EBF at enrollment	<6 months (day 3, weeks 6, 14 and 22)	43.7%<br>(22w; n=490)	Moderate -GRADE
35	Sepeng & Ballot, 2016	Retrospective record review	U (Johannesburg, GP)	404	Non-ICU records of neonates admitted to a BFHI tertiary hospital	Neonates within 72 hours of birth (at discharge)	23.3%<br>(<1w; n=404)	Med -aNOS

S1 Table. Summary of quantitative studies, longest EBF rates and quality outcome

36	Siziba et al, 2016	Cross-sectional	U/PU (NW, GP, FS, EC)	580	Mothers/caregivers of infants <6m accessing one of 40 high-volume health facilities selected from the four provinces	<6 months	12.0%<br>(6m; n=34)	Medium<br>-aNOS
37	Steyn et al, 2017	Cross-sectional	U (Cape Town, WC)	104	Mothers attending International Board Certified Lactation Consultant private practices	Not stated	55.8%<br>(n/s; n=104)	Low<br>-aNOS
38	Thomas et al, 2017	Cohort	PU (Drakenstein, WC)	720<br>*(for multivariable analysis)*	Mother-infant dyads from 2 primary health care clinics:<br>Mbwekeni (black African) and TC Newman (mixed ancestry) patients	Up to 24 months (6–10 weeks, 14 weeks, 6 months)	% not given<br>(<6m; n=720)<br><br>*Mean duration 2m (IQR 1-4)*	Medium<br>-aNOS
39	Tuthill et al, 2017	Randomised controlled trial	T (Sweetwaters and Edendale, KZN)	58<br>*29 Study*<br>*29 Control*	Mothers (age 18+) HIV+ using ART attending one of the 2 clinics	6 weeks	81.5%<br>(6w; n=58)	Moderate<br>-GRADE
40	Tuthill et al, 2017	Retrospective cohort	T (Sweetwaters and Edendale, KZN)	58	Mothers (age 18+) HIV+ using ART attending one of the 2 clinics	6 weeks	81.5%<br>(6w; n=58)	Low<br>-aNOS
41	Yako & Nzama, 2013	Cross-sectional	T (Mdantsane, EC)	60	Mothers attending their 6 week postnatal visit at a community health clinic PMTCT programme, irrespective of HIV status	~6 weeks	13.3%<br>(6w; n=60)	Medium<br>-aNOS
* R=rural; PU/T= peri-urban/ township; U=urban<br>** Only the longest durations and/or strongest measures reported for each article								
