# Supplementary material for: Exclusive breastfeeding policy, practice and influences in South Africa, 1980 to 2018: A mixed-methods systematic review
Source: PLoS One. 2019 Oct 18;14(10):e0224029. doi: 10.1371/journal.pone.0224029 (PMC6799928; doi:10.1371/journal.pone.0224029)
Supplement: S2 Table — (PDF) [file pone.0224029.s004.pdf]

## S2 Table. Observational evidence on EBF barriers (-) and facilitators (+)

| **Levels and factors** | **Article references for *significant (p<0.05)* findings [from S1 Table]** | **Notes on evidence** |
|---|---|---|
| **Individual level** | | |
| Mother attributes | | Until recently, HIV has been linked to EFF (vs. EBF), but that may be changing. Recent studies are finding both positive and negative associations with HIV status. Linked to HIV, fear of transmission influences decisions, while time of diagnosis may play a role in avoiding EBF. Health concerns may support EBF, except when milk insufficiency is perceived, which was consistently a barrier to EBF over time. Being employed or in school were two of the greatest barriers to EBF. Knowledge about EBF, positive attitudes and intention were associated with EBF in a few studies, although many studies looking at these variables did not find associations. Data on marital status is unclear. Higher SES (and related indictors) has also been noted as a barrier, although not in all cases. |
| HIV status of mother (+/-) | [12,16,17,19,26,29,35, 36,38,41] | |
| *Fear of HIV transmission (+/-)* | *[17,19,23,26]* | |
| *Pre-pregnancy HIV diagnosis (-)* | *[38]* | |
| Infant health concerns (+) | [17,18,20] | |
| *Milk insufficiency beliefs (-)* | *[1,6,8,9,10,15,17,20,33, 36,37]* | |
| Employment (-) | [6,8,10,17,19,20,25,34, 36,38,41] | |
| In school (-) | [8,17,20,33,36,41] | |
| Urban (-/+) | [5,13,34] | |
| Completed education (-) | [1,14] | |
| Higher SES (-) | [33,36] | |
| *Own fridge (-)* | *[13,17]* | |
| *Electricity, gas, parrafin (-)* | *[12,13]* | |
| EBF knowledge (+) | [3, 19, 32] | |
| Positive breastfeeding attitudes (+) | [2] | |
| Breastfeeding intention (+) | [1,4,12] | |
| Married (-/+) | [25,32,38] | |
| Older age of mother (+) | [25,26,41] | |
| Higher parity (-/+) | [25,37] | |
| Delivery by c-section (-/+) | [15,34] | |
| Mother ill (-) | [1,8,12] | |
| Past breastfeeding experience (+) | [34] | |
| EBF emotionally challenging (-) | [37] | |
| Low confidence/self-efficacy (-) | [15,41] | |
| Prenatal depression | [40] | |
| Living with infants grandmother (+) | [40] | |
| Alcohol use (-) | [17] | |
| Infant attributes | | |
| Infant age (-) | [8,18,22,29,32,37] | As infants get older, EBF reduces. Infants who actively refused breastmilk or who exhibited discomfort, usually described as crying or thirst, were more likely to be fed complementary foods. |
| Refusal of breastmilk (-) | [20,36,37] | |
| Colic/constipation (-) | [1,21,41] | |
| Crying (-) | [29] | |
| Recusitatated at birth (-) | [35] | |
| Birth asphyxia (-) | [35] | |
| Perceived to be hungry/thirsty (-) | [36] | |
| Growth (+) | [20] | |
| Mother-infant relationship | | |
| Breast problems, e.g. mastitis, flat nipples, pain (-) | [1,6,8,10,13,15,17,20,36 ,37,41] | Breast problems were a major reason for stopping EBF. |
| Feeding problems, poor latching (-) | [10,13,15] | |

## S2 Table. Observational evidence on EBF barriers (-) and facilitators (+)

| | | |
|---|---|---|
| **Settings level** | | |
| Health systems and services<br>Medical advice (+/-)<br>Postnatal support/visits (+)<br>Free formula programme (-)<br>Prelacteal feeds (-)<br>Separation of mother & infant (-)<br>District hospital delivery (+) | <br>[1,3,8,15,18,36,37]<br>[6,7,8,12,34]<br>[13,19,23]<br>[4,10]<br>[4,14]<br>[1] | The advice mothers received from health workers is important, and can support or undermine EBF. Postnatal support was positive, whereas hospital practices and the free formula programme undermined EBF. |
| Family & Community<br>Family advice (-/+)<br>Shared caregiving (-) | <br>[1,3,17,18,19,32,36,41]<br>[20] | Families were influential, but not consistently in support of EBF. |
| Workplace & School<br>-Maternity leave vs. none (-)<br>(See employment/school at individual level) | <br>[3] | Counterintuitively, having maternity leave was associated with returning to work earlier in one case. |
| **Structural level** | | |
| Socio-cultural context<br>Infant cleaning rituals/practices, e.g. use of muthi (-)<br>Dirty milk – expressing (-)<br>Unspecified cultural factors (-) | <br>[1,18,20]<br><br>[22]<br>[34] | Socio-cultural factors were consistently reported as barriers to EBF. |
| Market context | | No studies measured mass media exposure |
