# Supplementary material for: Exclusive breastfeeding policy, practice and influences in South Africa, 1980 to 2018: A mixed-methods systematic review
Source: PLoS One. 2019 Oct 18;14(10):e0224029. doi: 10.1371/journal.pone.0224029 (PMC6799928; doi:10.1371/journal.pone.0224029)
Supplement: S3 Table — (PDF) [file pone.0224029.s005.pdf]

S3 Table. Qualitative article summary table, by policy period

#	Article Ref	Study Design	Setting (R=rural; PU/T= peri-urban/ township; U=urban)	Sample Characteristics	Data Collection Methods	Infant Age	Analysis
**Period 1 (1980-1999)**							
1	Kruger & Gericke, 2001	Mixed methods	R (Moretele, NW)	154 caregivers<br>*-24 caregivers of infants <6m*	24 FGDs<br>*-4 FGDs*	0-36 months	Ethnographic content analysis
2	Seidel, 2000	Sociological, informed by discursive theory & feminist scholarship	U (Pietermaritzburg, KZN)	Healthcare workers	IDIs (semi-structured & open)	Not relevant	Social constructionism
3	Seidel, 2004	Sociological, using ethnographic and multiple qualitative methods<br><br>Critical & discursive theory, feminist	R & U (Sundumbili,<br><br>Songonzima<br><br>Songonzima, Edendale & Pietermarizburg, KZN)	120 multiparous mothers<br><br>Traditional birth attendants (TBAs)<br><br>Health workers, AIDS counsellors & doctors	IDIs (semi-structured & open)<br>Group discussions<br><br>Interviews	Not stated	Discursive
4	Seidel et al., 2000	Sociological	U (Durban, KZN)	13 HIV+ mothers who attended a peer support group	2 in-depth discussions, including role play	Not stated (some dead)	Social constructionism

S3 Table. Qualitative article summary table, by policy period

**Period 2 (2000-2007)**							
5	Buskens & Jaffe, 2008	Ethnographic	R (Watersmeet, KZN, Idutwa, EC)<br>PU/T (Soweto, GP; Langa & Hanover Park, WC, Motherwell, EC)<br>U: (Ladysmith, KZN, Ravonsmead, WC)<br>*+ Namibia & Swaziland*	82 HIV+ mothers in PMTCT<br>2 pregnant women<br>15 relatives<br>7 counsellors<br>7 nurses<br>3 PMTCT coordinators	In/formal interviews;<br>7 FGDs;<br>Observations	Not specified	Conceptual framework analysis and analytic retroduction
6	Buskens, Jaffe, & Mkhatshwa, 2007	Ethnographic	Same as above	Not disaggregated by country; likely same as above	In/formal interviews;<br>FGDs;<br>Observations	Not specified	Conceptual framework analysis
7	Chopra et al, 2002	Mixed methods, rapid assessment	PU/T (Khayelitsha, WC)	11 HIV+ mothers<br>11 counsellors	IDIs (structured)	Not specified	Content analysis
8	Doherty et al, 2006 (a)	Qualitative	R (Rietvlei, EC)<br>PU/T (Paarl, WC, Umlazi, KZN)	40 HIV+ mothers<br><br>Community health workers (CHWs)	40 IDIs (semi-structured)<br>3 FGDs (8-10 in each)	8 months (mean age)	Thematic content
9	Doherty et al, 2006 (b)	Qualitative, longitudinal	Same as above	27 HIV+ mothers at pregnancy then, 1, 4, 6 12 weeks postpartum	116 IDIs	12 weeks	Thematic analysis

S3 Table. Qualitative article summary table, by policy period

10	Du Plessis, 2009	Mixed methods	U (Johannesburg, GP)	6 primigravidaes attending new mother support group at private clinic	6 IDIs	>6 weeks	Descriptive analysis
11	Hildebrand, Goemaere & Coetzee, 2003	Mixed methods	PU/T (Khayelitsha, WC)	38 HIV+ mothers in a PMTCT programme	FGDs	12 weeks mean age	Not specified
12	Mackowski, 2005 [MA Thesis]	Qualitative	PU/T (Khayelitsha, WC)	5 HIV+ mothers in the m2m programme<br>3 mentor mothers<br>3 site coordinators	11 IDIs (semi-structured)	3-9 months	Grounded theory approach
13	Nor et al, 2009	Qualitative	R (EC)<br>PU/T (WC)<br>PU/T (KZN)	27 mothers	17 IDIs<br>10 Observations	12-24 weeks	Not specified
14	Nor et al, 2011	Qualitative, emergent inductive design	See above	17 HIV+ and HIV- mothers	17 IDIs (semi-structured)	12-16 weeks	Qualitative interpretive description & social constructivist conceptual framework
15	Sibeko et al., 2009	Qualitative	PU/T (KZN)	11 mothers (HIV+ and HIV-)	IDIs	3 months (avg.)	Content analysis
16	Stinson & Myer, 2012	Not stated [Qualitative]	U (Cape Town, WC)	11 HIV+ ≤ 6-month postpartum<br>*17 HIV+ pregnant*	IDIs	<6 months	Thematic approach to narrative analysis

S3 Table. Qualitative article summary table, by policy period

17	Thairu et al, 2005	Ethnographic nested in cohort study	R (KZN)	22 HIV+ mothers, part of cohort study	Ethnographic interviews (semi-structured)	Not stated	Conversation and thematic analysis
18	Varga & Brookes, 2008	Narrative research - Participatory	PU/T (Mankweng , LP)<br>R (Siloam, LP)	10-15 teen mothers, clinic staff, VCT counsellors & young adults<br><br>100 teen mothers (50 rural:50 urban)<br><br>Teen (15-19 year old) mothers	2 Key informant workshops<br><br>Community-based survey<br><br>2 FGDs (10-12 each)	Not stated	Grounded theory approach
**Period 3 (2008-2011)**							
19	Andreson et al, 2013	Mixed methods, longitudinal	R (Butterworth & Nozuko, EC)	12 HIV+ mothers<br>12 "Buddies"	IDIs (3x)<br>IDIs (3x)	2-6 months by end	Content analysis
20	Chaponda, Goon & Hoque, 2017	Qualitative	U (Thembisa, GP)	30 HIV+ mothers in postnatal wards	IDIs<br>Observations	Not specified	Thematic analysis
21	Goosen, McLachlan & Schübl, 2014 (a)	Mixed methods	PU/T (Avian Park & Zwelenthemba, WC)	65 mothers<br>*-17 EBF/predom.*<br>*-19 partial BF*<br>*-29 no BF*<br>26 fathers<br>20 grandmothers<br>9 healthcare workers (HCWs)	7 FGDs<br>*-2 FGDs*<br>*-2 FGDs*<br>*-3 FGDs*<br>3 FGDs<br>2 FGDs<br>1 FGD	<6 months	Thematic

S3 Table. Qualitative article summary table, by policy period

22	Goosen, McLachlan & Schübl, 2014 (b)	Qualitative	See above	36 mothers<br>*-17 EBF/predom.*<br>*-19 partial BF*<br>26 fathers<br>20 grandmothers<br>9 HCWs	4 FGDs<br>*-2 FGDs*<br>*-2 FGDs*<br>3 FGDs<br>2 FGDs<br>1 FGD	See above	Content analysis
23	Ijumba et al, 2012<br>*[NB: all formula feeding mothers]*	Qualitative	PU/T (KZN)	11 HIV+ mothers 9 HIV- mothers<br>14 grandmothers<br>13 fathers<br>13 teen mothers	11 IDIs<br>9 IDIs<br>2 FGDs (7;7)<br>2 FGDs (7; 6)<br>FGD HIV+ (7)<br>FGD HIV- (6)	<6 months	Thematic analysis
24	Laher et al, 2012	Mixed methods, concurrent	PU/T (Soweto, GP)	45 HIV+ mothers of HIV+ infants in PMTCT programme	35 structured interviews<br>2 FGDs (10)	Not stated	Thematic and content analysis
25	Madiba & Langa, 2014	Qualitative 2011-2012	U/T (Tshwane, GP)	43 HIV+ mothers	5 FGDs	3-6 months	Thematic data analysis
26	Madiba & Letsoalo, 2013	Not stated [Qualitative]	U/T (Tshwane, GP)	25 HIV+ mothers in PTMCT programme	4 FGDs	6 weeks to 6 months	Thematic data analysis
27	Ramara, Maputle & Lekhuleni, 2010	Phenomen-Ological	R (Mankweng, LP)	10 HIV+ mothers	30 IDIs (3x)	2-24 weeks	Tesch's open-coding
28	Swarts, Kruger & Dolman, 2010<br>**Unclear sample size*	Not stated [Mixed methods]	R (Lower Umfolozi District, KZN)	22 mothers staying at hospital lodge (infants had complications)	4 FGDs (8 each), but only 3 used b/c data saturation*	Not stated	"Coded by topic"

S3 Table. Qualitative article summary table, by policy period

29	Zulliger, Abrams, & Myer, 2013	Mixed methods, convergent parallel	PU/T (Gugulethu, WC)	12 HIV+ mothers (out of 34, which included pregnant)	IDIs (semi-structured)	"Recent postpartum"	Open-coding and Thematic
**Period 4 (2012-2018)**							
30	Hunter-Adams et al, 2016	Qualitative	U (Cape Town, WC)	23 Congolese, Somali and Zimbabwean mothers<br>21 men and 27 women from three countries	IDIs<br><br>9 FGDs (3 male & 6 female, by origin)	Mothers had to have given birth in past 2 years	Thematic analysis
31	Jama et al, 2017	Qualitative, longitudinal cohort	R (KZN)<br>U (KZN)	22 mothers (including some teenagers 15-19, HIV+ and working) who intended to EBF	125 IDIs	<6 months	Framework analysis
32	Modiba, 2015<br><br>*[NB: all formula feeding mothers]*	Qualitative, exploratory, descriptive & contextual	U (GP)	12 HIV+ mothers enrolled at least 3 months in PMTCT Programme	IDIs (semi-structured)	Not stated	Thematic content
33	Mushaphi et al., 2017	Nested qualitative in cohort study	R (Dzimauli, LP)	37 caregivers	4 FGDs (7-10 each)	6 months	Thematic content analysis
34	Ntuli & Modibedi, 2015	Qualitative, exploratory	U (Tshwane, GP)	32 HIV+ mothers in PMTCT programme	5 FGDs (6-8 each)	6 weeks to 6 months	Thematic content analysis & open coding
