# Supplementary material for: Exclusive breastfeeding policy, practice and influences in South Africa, 1980 to 2018: A mixed-methods systematic review
Source: PLoS One. 2019 Oct 18;14(10):e0224029. doi: 10.1371/journal.pone.0224029 (PMC6799928; doi:10.1371/journal.pone.0224029)
Supplement: S4 Table — (PDF) [file pone.0224029.s006.pdf]

S4 Table. Qualitative Meta-synthesis of themes and sub-themes

Levels and factors	Article references [Linked to S3 Table]
**INDIVIDUAL LEVEL**	
MOTHER ATTRIBUTES<br>**Mother HIV positive status:** Being HIV positive has influenced feeding choices and exclusivity across policy periods, mostly towards EFF but recently EBF:<br>***Fear of HIV transmission to the infant*** was discussed mostly around mixed feeding, although fears of any breastmilk were expressed consistently<br>***Knowledge about transmission risk*** in breastmilk and while on ART tended to be inaccurate, usually overestimating risk (except early on)<br>***Fear of disclosure through feeding method (formula and/or exclusivity)***, based on perceived community norms (see Community)<br>***Timing of HIV diagnosis***	 [2, 3, 4, 5, 6, 7, 8, 9, 11, 12, 14, 15, 16, 17,19,20, 21, 23, 24, 25, 27, 28, 31]<br>*[4, 5, 6, 7, 8, 9, 11, 12, 14, 15, 16, 17,19, 20, 21, 23, 25, 27, 28, 29, 31, 32, 34]*<br>*[2, 3, 4, 5, 6, 7, 8, 9, 11, 12, 14, 15, 17, 19, 20, 25, 27, 32]*
**Employment:** The need to find work and/or go to work, resulted in formula feeds<br>***Unemployment:*** While generally supporting EBF, financial dependence also limited perceived choice	[2, 3, 4, 7, 9, 11, 22, 28, 29, 30, 31, 33]<br>*[1, 2, 3, 7, 11, 17, 22, 23,25]*
**Young age/Adolescent:** Young mothers talked about having less power to decide on feeding choices (see also Household). Some age-specific barriers to EBF:<br>***Return to school,*** resulting in a shift to formula<br>***Body image***, such as sagging breasts due to breastfeeding	[17,23]<br><br>*[2, 7, 23, 28, 31, 33]*<br>*[2, 4, 23, 29]*
**Past feeding experiences:** Depending on the outcomes of experiences, this could influence current choices	[3, 4, 5, 6, 7, 12, 13, 29, 30, 34]
**Breastfeeding knowledge:** Accurate knowledge assisted EBF, particularly:<br>***Milk sufficiency**:* Knowing that breastmilk was enough for an infant<br>***Duration:*** Knowing how long to breastfeed exclusively<br>***Superiority:*** Being able to cite the superiority of breastmilk over formula<br>***Low cost:*** Avoidance of formula costs supported breastfeeding choice	 [1, 2, 4, 5, 6, 7, 8, 9, 11, 12, 19, 20]<br>*[4, 5, 6, 11, 12, 14, 15, 19, 20, 22,27, 29, 30]*<br>*[5, 10, 12, 14, 20]*<br>*[1, 2, 3, 4, 6, 8, 17, 25, 28, 29, 31, 33]*

S4 Table. Qualitative Meta-synthesis of themes and sub-themes

	[3, 11, 13, 19, 20, 25, 28]
**Contamination beliefs:** Ideas that social or behavioural interactions could taint the quality of breastmilk and be passed onto infants threatened EBF	[2, 3, 11, 22]
**Self-efficacy/confidence to breastfeed:** When low, this was also a threat to EBF	[7, 8, 12, 31]
**Expressing:** Willingness and ability to express breastmilk supported EBF	[3, 31]
<u>INFANT ATTRIBUTES</u>	
**Temperment, especially crying:** Crying was often interpreted as hunger or discomfort, leading to mixed feeding. Feeding was also used to silence crying.	[1, 3, 4, 7, 12, 14, 15, 19, 21, 22, 27, 30, 31, 33]
**Growth:** Mothers used growth to identify if feeding choices were appropriate.	[1, 4, 5, 6, 14, 15, 19, 22, 28, 30]
**Health events:** Conditions like oral thrush or HIV conversion led to feeding changes, usually away from EBF	[3, 4, 9, 10, 11, 15]
<u>MOTHER-INFANT RELATIONSHIP</u>	
**Current feeding experiences:** The way feeding was experienced by mothers influenced whether feeding choices were maintained or not	[1, 3, 4, 5, 6, 9, 10, 11, 12, 13, 14, 20, 29, 31]
Practicality/ease: How well a feeding choice was perceived to fit with lifestyle	*[1, 3, 6, 9, 12, 20, 23, 25, 28, 29, 30, 31, 33, 34]*
Percieved sufficiency: Whether mothers felt their infant was satiated by milk	*[1, 3, 4, 5, 6, 7, 9, 10, 11, 14, 15, 20, 30, 31, 34]*
Bonding/love: Mothers feeling closer to their infants supported EBF	*[3, 5, 6, 11, 12, 14, 28]*
Breast problems: Mastitis, engorgement, and cracked nipples hindered EBF	*[1, 3, 6, 10, 11, 12]*
Food insecurity: Mothers felt their own food insecurity contributed to insufficient milk, which led to mixed feeding	*[6, 30]*
**Latching:** When infants struggled to latch, breastfeeding was abandoned	[10, 32]

S4 Table. Qualitative Meta-synthesis of themes and sub-themes

**SETTINGS LEVEL**	
HEALTH SYSTEMS AND SERVICES<br>**Medical advice/counselling:** Health workers were reported to influence feeding choices, both for or against EBF, based on their advice and the following:<br>***(In)consistency of messages:*** Within-facility inconsistencies were particularly damaging to EBF, often noted between PMTCT and labour wards<br>***(In)adequate messages:*** Mothers often felt there were not told things that would have assisted them to make a choice or prepare for challenges<br>***EBF only:*** If clear, single messages supported EBF, but messages were also experienced as inconsistent with community knowledge and/or experiences<br><br>**Free formula programme:** This reduced EBF practices and had its own issues:<br>***Insufficient/stock-outs:*** Mothers using the programme reported not having enough to feed their infants and resorting to mixed feeding<br>***Brand stigma:*** Associations between Pelargon formula from government and HIV meant mothers tried to buy alternatives or hide brand<br><br>**Mother-Baby Friendly Hospitals:** These facilities were noted by mothers, often by the forceful attitudes of staff as well as pro-breastfeeding practices:<br>***Restricting bottles or formula:*** This was often described in the context of mothers hiding their feeding practices or being "forced" to change practices<br>***Lack of separation***, where mothers were able to stay with infants helped breastfeeding, although not all MBFH's followed through<br>***Latching support***, provided by health workers was highly appreciated	[1, 2, 3, 4, 5, 7, 8, 9, 10, 12, 14, 15,17, 20, 22, 24, 25, 27, 28, 29, 31, 32, 33, 34]<br>*[3, 4, 5, 7, 8, 9, 10, 12, 14, 15, 20, 24, 27, 29, 31, 32]*<br>*[1, 2, 3, 4, 5, 7, 10, 12, 14, 15, 17,20, 22, 24, 29, 31]*<br>*[3, 4, 5, 14, 20, 25, 29, 31, 34]*<br><br>[3, 4, 5, 6, 8, 9, 12, 14, 15, 16, 19, 20, 24, 25, 28]<br>*[4, 6, 7, 8, 9, 12, 14, 15, 17,19, 24, 25]*<br>*[12, 24,27, 29, 32]*<br><br>[2, 4, 5, 10, 12, 31, 34]<br>*[2, 4, 5, 10, 12, 34]*<br>*[9, 10, 31]*<br>*[31, 33]*
FAMILY SETTING<br>**Family influence:** Family members, particulary mothers, influence decisions. Fathers and siblings also influence. Specific influences included:<br>***Socio-cultural:*** Pressuring mothers to follow cultural/family practices of feeding or cleaning that threaten EBF<br>***Living/caregiving arrangements:*** Influenced decisions, giving family members more say over feeding practices, especially of young mothers	[1, 2, 3, 4, 5, 6, 8, 9, 10, 12, 14, 15, 17, 18, 19, 20, 22, 23, 24, 25, 26, 27, 29, 31, 32, 33, 34]<br>*[2, 3, 4, 5, 6, 8, 9, 12, 14, 15, 17, 18, 20, 22, 25, 26, 31, 33]*<br>*[2, 3, 4, 8, 9, 12, 15, 17, 20, 22, 23, 25,27,28,29]*

S4 Table. Qualitative Meta-synthesis of themes and sub-themes

Financial dependency: Families who financially supported mothers and infants had more say over feeding practices<br>**Family support for HIV positive mothers:** This was strongly linked to support for EBF if the mothers disclosed<br>***Threat of violence/abandonment:*** These fears worked against disclosure	*[2, 3, 6, 8, 9, 12, 14, 15, 20, 22, 23]*<br>[2, 4, 5, 6, 7, 8, 9, 12, 15, 17, 18, 19, 20, 23, 25,26,27]<br>*[2, 3, 4, 5, 6, 18, 27, 32]*
<u>WORKPLACE/SCHOOL SETTING</u><br>**Employee letters:** One mother talked about trying to influence an employer to support EBF through a letter from the clinic.	[27]
<u>COMMUNITY SETTING</u><br>**Gossiping/HIV stigma:** This was raised as an EBF barrier, as the practice is linked to HIV, which mothers addressed in the following ways:<br>***Concealment:*** Where mothers hid their true feeding practice to avoid stigma<br>***Explanation:*** Where community is educated about importance of EBF to gain support and to address/reduce HIV stigma<br><br>**Community support interventions:** Several articles described these directly:<br>**Buddies/peers/mentor mothers:** Mothers appreciated locally available support to advise or encourage them in the community setting<br>**Postnatal support:** A variety of ideas were suggested by mothers in addition to actual support through postnatal counselling, support groups and income generation projects.<br>**Milk bank/wet nurse:** This was noted as a support option only once	[3, 4, 5, 6, 7, 8, 12, 13, 14, 15, 17, 18, 24, 25, 26, 27, 28, 29, 32, 34]<br>*[3, 8, 12, 15, 17, 24, 25, 26, 27, 29, 31, 32, 34]*<br>*[5, 12, 19]*<br><br>[5, 11, 12, 13]<br>*[12, 13, 19]*<br><br>*[5, 7, 11, 12, 22, 31]*<br><br>*[3]*
**STRUCTURAL LEVEL**	
<u>SOCIO-CULTURAL CONTEXT</u><br>**Mixed feeding norms:** Expecations to mixed feed from family and community were consistently referenced as a challenge for EBF<br>***Breastfeeding norms:*** They expectation to breastfeed (often as part of mixed feeding) was noted by mothers across all policy periods	[1, 2, 3, 4, 5, 6, 7, 8, 9, 11, 12, 14, 15, 18, 23, 25, 26, 31, 33]<br>*[1, 2, 3, 4, 11, 12, 14, 15, 17, 32]*

S4 Table. Qualitative Meta-synthesis of themes and sub-themes

**Motherhood norms:** Ideas of what it meant to be a good mother influenced decisions<br><br>**HIV stigma against formula feeding**, was reported widely<br><br>**HIV stigma towards any sort of exclusive feeding** was also reported<br><br>**HIV stigma towards intervention**, such as buddies, was another experience	[2, 3, 4, 5, 6, 16, 31]<br><br>[2, 3, 4, 7, 8, 9, 11, 12, 14, 15, 16, 17, 19, 24, 25, 28, 29, 32]<br>[2, 3, 4, 5, 7, 8, 9, 14, 15, 34]<br><br>[12, 13]
MARKET CONTEXT<br>**Culture of commercial formula:** A few articles highlighted how use of commercial formula is perceived as a culture in South Africa, preferred above other complementary foods<br><br>**Breastfeeding promotion:** Mothers talked about how materials/campaigns promoting breastfeeding influenced their decisions, with a call for clear messages<br><br>**Media engagement:** Mothers discussed channels they use to seek out information on infant feeding via the Internet or social media	 [21, 22, 30]<br><br><br><br>[14, 15]<br><br><br>[33, 34]
